# DNA-PK triggers histone ubiquitination and signaling in response to DNA double-strand breaks produced during the repair of transcription-blocking topoisomerase I lesions

**DOI:** 10.1093/nar/gkv1196

**Published:** 2015-11-17

**Authors:** Agnese Cristini, Joon-Hyung Park, Giovanni Capranico, Gaëlle Legube, Gilles Favre, Olivier Sordet

**Affiliations:** 1 Cancer Research Center of Toulouse, INSERM UMR1037, Toulouse 31037, France; 2 Department of Pharmacy and Biotechnology, University of Bologna, Bologna 40126, Italy; 3 Université de Toulouse, UPS, LBCMCP, 31062 Toulouse, France; 4 CNRS, LBCMCP, 31062 Toulouse, France

## Abstract

Although defective repair of DNA double-strand breaks (DSBs) leads to neurodegenerative diseases, the processes underlying their production and signaling in non-replicating cells are largely unknown. Stabilized topoisomerase I cleavage complexes (Top1cc) by natural compounds or common DNA alterations are transcription-blocking lesions whose repair depends primarily on Top1 proteolysis and excision by tyrosyl–DNA phosphodiesterase-1 (TDP1). We previously reported that stabilized Top1cc produce transcription-dependent DSBs that activate ATM in neurons. Here, we use camptothecin (CPT)-treated serum-starved quiescent cells to induce transcription-blocking Top1cc and show that those DSBs are generated during Top1cc repair from Top1 peptide-linked DNA single-strand breaks generated after Top1 proteolysis and before excision by TDP1. Following DSB induction, ATM activates DNA-PK whose inhibition suppresses H2AX and H2A ubiquitination and the later assembly of activated ATM into nuclear foci. Inhibition of DNA-PK also reduces Top1 ubiquitination and proteolysis as well as resumption of RNA synthesis suggesting that DSB signaling further enhances Top1cc repair. Finally, we show that co-transcriptional DSBs kill quiescent cells. Together, these new findings reveal that DSB production and signaling by transcription-blocking Top1 lesions impact on non-replicating cell fate and provide insights on the molecular pathogenesis of neurodegenerative diseases such as SCAN1 and AT syndromes, which are caused by TDP1 and ATM deficiency, respectively.

## INTRODUCTION

Topoisomerase I (Top1) is required to remove DNA supercoiling generated during transcription. It relaxes DNA by producing transient Top1 cleavage complexes (Top1cc), which are Top1-linked DNA single-strand breaks (SSB) (1). After DNA relaxation, Top1cc reverse rapidly and Top1 is released as the DNA religates. Top1cc can be trapped under a broad range of physiological conditions including oxidative base damages, alkylation by carcinogenic compounds and nicks (see Table 1 in reference (2)), and by ribonucleotide misincorporation (3–5). Top1cc can also be trapped selectively by camptothecin (CPT) and its derivatives used to treat cancers, which bind at the Top1-DNA interface (1). Stabilized Top1cc are potent transcription-blocking DNA lesions (6,7) and their repair (removal) depends primarily on the tyrosyl–DNA phosphodiesterase-1 (TDP1) excision pathway. Top1cc excision by TDP1 requires prior proteolysis of Top1 by the ubiquitin/proteasome system (2,8–14). Defective repair of Top1cc by inactivating mutation of TDP1 leads to the hereditary spinocerebellar ataxia with axonal neuropathy-1 (SCAN1) syndrome (15,16), indicating the importance of removing transcription-blocking Top1cc in non-replicating cells. A consequence of transcription-blocking Top1cc is the production of DSBs. These co-transcriptional DSBs have been detected in post-mitotic neurons and lymphocytes as well as in replicating cells out of the S-phase (17–19). Their production involves the formation of R-loops, a three-strand nucleic acid structure consisting of an RNA:DNA hybrid and displaced single-stranded DNA (20,21). Whether the Top1cc repair process is involved in the production of co-transcriptional DSBs is an unresolved question.

DNA double-strand breaks (DSBs) are among the most severe genomic lesions, and their repair requires the recruitment of DNA damage response (DDR) proteins in the vicinity of damaged chromatin, where they form discrete nuclear foci (22). The serine/threonine kinase ATM is critical for DDR (23) and its deficiency leads to the hereditary ataxia telangiectasia (AT) syndrome, which is primarily a neurodegenerative disease (15,24). ATM is readily activated by DSBs and phosphorylates various DDR proteins at damaged sites such as histone H2AX and MDC1. Phosphorylated H2AX (known as γH2AX) binds MDC1, which amplifies the damage signal around the break by recruiting additional ATM molecules (23). Accumulating studies indicate that histone ubiquitination regulates DDR both upstream and downstream of ATM. Ubiquitination of H2AX by the E3 ligase activity of RNF2–BMI1 complex triggers recruitment of activated ATM to DSBs allowing ATM to phosphorylate its targets at damaged sites (25,26). Then, ATM-mediated phosphorylation of MDC1 provides a binding site for the E3 ligase RNF8, which permits the recruitment of the E3 ligase RNF168. The concerted action of RNF8 and RNF168 allows ubiquitination of H2AX and H2A leading to the further recruitment of repair proteins such as 53BP1 and the BRCA1 complex (27–32). DNA-PK is also rapidly recruited at DSBs where it mediates repair by non-homologous end-joining (NHEJ) (33). Although DNA-PK can phosphorylate H2AX in response to DSBs (34), it is not clear whether it participates to DDR signaling asides from its role in DSB repair.

Here, we use serum-starved quiescent cells treated with CPT as a model to induce specifically transcription-blocking Top1cc and get molecular insights into the processes underlying both the production and signaling of DSBs. We found that those DSBs are produced during Top1cc repair from Top1 peptide-linked DNA SSBs generated after Top1 proteolysis and before excision by TDP1. These data provide the first demonstration that TDP1, whose deficiency leads to neurodegeneration, protects non-cycling cells against the formation of DSBs. Analysis of DSB signaling further reveals a novel function of DNA-PK in promoting protein ubiquitination leading to enhancement of Top1 proteolysis in a feedback loop as well as to full ATM activity at DSB sites. Lastly, we found that those co-transcriptional DSBs kill quiescent cells indicating that the cellular response to transcription-blocking Top1 lesions impact on non-proliferative cell fate. Together, these findings provide new insights on the molecular pathogenesis of neurodegenerative diseases.

## MATERIALS AND METHODS

### Drugs and chemical reagents

BrdU, CPT, FLV, MG132, Pyr-41 (35) and 4-hydroxitamoxifen (4OHT) were obtained from Sigma-Aldrich; lactacystin, G5 (36), KU55933 (37) and VE-821 (38) from Millipore; bortezomib, veliparib and olaparib from Selleckchem; and NU7441 (39) from Tocris. BrdU was dissolved in water, 4OHT in ethanol and the other agents in DMSO.

### Cell lines, culture and treatments

Primary human lung embryonic WI38 fibroblasts immortalized with hTERT were obtained from Estelle Nicolas (LBCMCP, Toulouse, France) and Carl Mann (CEA, Gif-sur-Yvette, France) (40). Cells were cultured in modified Eagle's medium (MEM) supplemented with 10% (v/v) fetal bovine serum, 1 mM sodium pyruvate, 2 mM glutamine and 0.1 mM non-essential amino acids. Normal human dermal fibroblast (NHDF) cells were isolated from healthy patients, as described previously (41), and human primary lung IMR90 fibroblasts were from ATCC. NHDF and IMR90 cells were cultured in Dulbecco's modified Eagle's medium (DMEM) supplemented with 10% (v/v) fetal bovine serum. To induce quiescence, cells were washed twice with serum-free medium and cultured for 72 h in medium supplemented as described above but with 0.2% (v/v) serum instead of 10%. U2OS EV28 cells, stably expressing *Asi*SI-ER-HA enzyme (42) were grown in DMEM supplemented with 10% (v/v) fetal bovine serum and 1 μg/ml puromycine. To induce DSBs, U2OS EV28 cells were treated with 300 nM 4OHT for 4 h. In Figure 2D and E, cells were gamma-irradiated with Gamma-cell 40 Exactor at 0.8 Gy. In all the experiments, mock samples were treated with the vehicle only as indicated.

### Immunofluorescence microscopy, foci quantification and graphical representation

Cells were seeded in poly-L-lysine-coated Lab-Tek^TM^ RS chamber slides (ThermoFisher). After treatment, cells were washed twice with PBS and immunofluorescence was carried out as described previously (17). Where indicated, cells were pre-extracted with CSK buffer (10 mM Pipes (pH 6.8), 100 mM NaCl, 300 mM sucrose, 3 mM MgCl_2_, 0.5% (v/v) Triton X-100) for 3 min at room temperature. Following two washes in PBS, cells were fixed with 2% (v/v) formaldehyde for 12 min at room temperature and washed three times. Cells were incubated with the primary antibody in PBS with 5% (v/v) fetal bovine serum for 1 h. Anti-ubiquityl-H2A antibody was incubated overnight at 4°C. Cells were washed twice and incubated with the appropriate secondary antibody coupled to Alexa Fluor 488, 594 or 568 (Life Technologies). After three washes, slides were mounted using Mowiol^®^ 4–88 (Millipore) containing 4′,6-diamidino-2-phenylindole (DAPI). Slides were visualized at room temperature by using a fluorescence microscope (Eclipse 90i; Nikon) or an inverted confocal microscope (LSM 710 or LSM 780; Carl Zeiss). Pictures were analyzed with Photoshop CS3 (Adobe) or ImageJ (version 1.48v). Primary antibodies used for microscopy were rabbit anti-53BP1 (NB100–305; Novus), rabbit anti-53BP1-pS1778 (2675; Cell Signaling), mouse anti-ATM-pS1981 (4526; Cell Signaling), mouse anti-BrdU (clone B44; BD Biosciences), mouse anti-γH2AX (05–636; Millipore), rabbit anti-γH2AX (NB100–384; Novus), mouse anti-DNA-PK-pT2609 (ab18356; Abcam), rabbit anti-MDC1 (ab11169; Abcam), mouse anti-ubiquityl-H2A (Ub-H2A; 05–678; Millipore) and mouse anti-ubiquitinated proteins (Ub-proteins; clone FK2, 04–263; Millipore).

Nuclear foci were counted manually and for nuclei with more than 20 foci, we set a default value of 25 foci. For Figures 2C and 3, nuclear foci were counted with ImageJ (version 1.48v). For graphical representation of foci distribution, we used box-and-whisker plots using GraphPad Prism 6 software with the following settings: boxes: 25–75 percentile range; whiskers: 10–90 percentile range; horizontal bars: median number of γH2AX foci. Each dot indicates an individual nucleus that is not in the 10–90 percentile range.

### Cell extracts and immunoblotting

Whole cell extracts were obtained by lysing cells in 1% SDS and 10 mM Tris-HCl (pH 7.4) supplemented with protease (Sigma-Aldrich) and phosphatase (Halt phosphatase inhibitor cocktail; ThermoFisher) inhibitors. Viscosity of the samples was reduced by brief sonication. For detection of ATM, ATM-pS1981, DNA-PK and DNA-PK-pS2056, cells were lysed for 15 min in buffer containing 50 mM Tris-HCl (pH 8.0), 300 mM NaCl, 0.4% (v/v) NP-40, 10 mM MgCl_2_ and 5 mM DTT, supplemented with protease (Sigma-Aldrich) and phosphatase (Halt phosphatase inhibitor cocktail; ThermoFisher) inhibitors. After centrifugation (10 000 x g, 20 min), supernatants were diluted (v/v) in 50 mM Tris–HCl (pH 8.0), 0.4% (v/v) NP-40 and 5 mM DTT. Proteins were separated by SDS-PAGE and immunoblotted with the following antibodies: anti-actin (MAB1501; Millipore), anti-ATM (sc-23921; Santa-Cruz), anti-ATM-pS1981 (ab81292; Abcam), anti-ATR (ab2905; Abcam), anti-caspase-3 (9662; Cell Signaling), anti-cleaved caspase-3 (9661; Cell Signaling), anti-Chk2 (2662; Cell Signaling), anti-Chk2-pT68 (2661; Cell Signaling), anti-cullin 3 (ab108407; Abcam), anti-cullin 4B (A303–863A; Bethyl), anti-DNA-PK (NA57; Millipore, or ab1832; Abcam), anti-DNA-PK-pS2056 (ab18192; Abcam), anti-γH2AX (NB100–384; Novus), anti-H2AX (ab11175; Abcam), anti-histone H3 (ab1791; Abcam), anti-KAP1 (A300–274A; Bethyl), anti-KAP1-pS824 (A300–767A; Bethyl), anti-PARP (9542; Cell Signaling), anti-PSMA6 (2459; Cell Signaling), anti-p53 (DO-7; DakoCytomation), anti-p53-pS15 (9284; Cell Signaling), anti-topoisomerase I (ab109374; Abcam), anti-αTubulin (T5168; Sigma-Aldrich), anti-UBA1 (4890; Cell Signaling), anti-XLF (2854; Cell Signaling) and anti-XRCC4 (from Patrick Calsou, IPBS, Toulouse, France). Immunoblotting was revealed by chemiluminescence using autoradiography or a ChemiDoc MP System (Bio-Rad). Quantification of protein levels was done by using ImageJ (version 1.48v) in Figure 4E and with Image Lab software (version 4.1) in the other figures.

### Top1 ubiquitination

Top1 ubiquitination was detected from whole cell extracts as described previously (43) with some modifications. Briefly, cells were lysed for 30 min on ice in IP buffer (50 mM Tris–HCl (pH 7.4), 150 mM NaCl, 2 mM EDTA, 2 mM EGTA, 0.2% (v/v) Triton X-100, 0.3% (v/v) NP-40, 20 mM *N*-Ethylmaleimide) supplemented with protease (Complete; Roche) and phosphatase (25 mM NaF, 25 mM β-glycerophoshate, 0.1 mM sodium orthovanadate) inhibitors. Cell lysates were sonicated (intensity settings: high, 4 cycles of 5 min, 30 s ON and 50 s OFF) at 4°C by using Bioruptor^®^ Standard (Diagenode) and then pre-cleared with 10 μl protein-A agarose beads (Repligen) for 1 h at 4°C. After centrifugation (1 min at 1000 x g), 2 mg of pre-cleared samples were subjected to immunoprecipitation with a rabbit anti-Top1 antibody (from Yves Pommier, NIH, USA) or a rabbit non-immune antibody (control IgG) (catalogue number 02-6102; ThermoFisher) overnight at 4°C followed by incubation with 10 μl protein-A agarose beads (Repligen) for 3 h at 4°C. After washing three times in IP buffer, beads were resuspended in 2X SDS sample buffer. Immunoprecipitates were separated on a 6% SDS-PAGE gel and immunoblotted with a mouse anti-ubiquitin antibody (clone P4D1; sc-8017; Santa-Cruz). The membrane was treated with Restore^TM^ PLUS Western Blot Stripping Buffer (ThermoFisher) for 10 min and re-probed with a rabbit anti-Top1 antibody (ab109374; Abcam).

### Cellular fractionation

Chromatin-bound proteins were isolated as described previously (44). Briefly, cells were extracted twice by lysis in extraction buffer (50 mM Hepes (pH 7.5), 150 mM NaCl, 1 mM EDTA) containing 0.1% (v/v) Triton X-100 supplemented with protease (Sigma-Aldrich) and phosphatase (Halt phosphatase inhibitor cocktail; ThermoFisher) inhibitors for 15 min at 4°C followed by centrifugation at 14 000 x g for 3 min to separate soluble proteins. The collected supernatant was the fraction S1. The pellet was further resuspended in extraction buffer without Triton X-100 supplemented with 200 μg/ml RNAse A (Roche) for 30 min at 25°C under agitation. The fraction S2 was collected after centrifugation at 14 000 x g for 3 min. The remaining pellet was resuspended in buffer containing 1% SDS and 10 mM Tris–HCl (pH 7.4) supplemented with protease (Sigma-Aldrich) and phosphatase (Halt phosphatase inhibitor cocktail; ThermoFisher) inhibitors and sonicated.

### Comet assays

Neutral Comet assays were performed according to the manufacturer's instructions (Trevigen), except that electrophoresis was performed at 4°C. Slides were visualized by using a fluorescence microscope (Eclipse 90i; Nikon, or AxioObserver Z1; Zeiss). Comet tail moments were measured with ImageJ software (version 1.48v) using a macro provided by Robert Bagnell (https://www.med.unc.edu/microscopy/resources/imagej-plugins-and-macros/comet-assay) or with the plugin OpenComet (http://opencomet.org/).

### Detection of Top1-DNA cleavage complexes

Cellular Top1-DNA cleavage complexes (Top1cc) were detected as previously described (45), except that immunoblotting was revealed with a rabbit monoclonal anti-Top1 antibody from Abcam (ab109374) and with a ChemiDoc MP System (Bio-Rad).

### Cell viability assays

Adherent cells were counted at times and conditions indicated in the text with Z1 Coulter Counter^®^ (Beckman Coulter). CellTiter Blue cell viability assays were performed into 96-well microplates according to the manufacturer's instructions (Promega) and fluorescence was measured at 590–20 nm Ex/620–15 nm Em using a Synergy^TM^2 Multi-Mode Microplate Reader (BioTek). Adhesion and viability of untreated cells was set to 100%.

### Chromatin immunoprecipitation (ChIP)

Chromatin immunoprecipitation (ChIP) was performed as described previously (42) using a rabbit monoclonal anti-γH2AX antibody (ab81299) or a rabbit nonimmune antibody (control IgG) (catalogue number 02-6102; ThermoFisher). ChIP were analyzed by real-time QPCR using primers proximal to two sites for the restriction enzyme *Asi*SI located inside genes (chr20:42089225–42089433: Gene 1-FW: AAAAGTCGCTCCCGGTAAAT, Gene 1-RV: CCGATCAGACTTGGGCTTAG; chr17:61847852–61848028: Gene 2-FW: TGCAAGGCATTCGACAATAA, Gene 2-RV: ATGGAAGCCATAATGCAAGC) or primers distal to *Asi*SI sites (chr21:25081874–25082075: Control-FW: TGGCTGGAACTGCTTTCTTT, Control-RV: GGTGAGTGAATGAGCTGCAA). All samples were analyzed in triplicates and data normalized to the maximal recovery in each experiment, which was set equal to 1.

### Quantification of global RNA transcription

Global RNA transcription was detected in cells using the Click-iT RNA Alexa Fluor 488 Imaging Kit (ThermoFisher). Cells were seeded in poly-L-lysine-coated Lab-Tek^TM^ RS chamber slides (ThermoFisher). For the last 30 min of each time point, cells were incubated with 1 mM 5-ethylnyl uridine (EU) to label newly synthesized RNA, which was detected according to the manufacturer's instructions. Slides were mounted using Mowiol^®^ 4–88 (Millipore) containing DAPI and visualized by using a fluorescent microscope (AxioObserver Z1; Zeiss). Pictures were analyzed with ImageJ (version 1.48v).

### Proteasome activity assay

P2 fractions were isolated as described above (see, Cellular fractionation). At each step of fractionation, the extraction buffer was supplemented with 10% (v/v) glycerol and 50 mM ATP without the addition of protease and phosphatase inhibitors. P2 fractions were solubilized in extraction buffer and briefly sonicated. Following centrifugation at 14 000 x g for 3 min, 30 μg of the collected supernatant were used to perform proteasome activity assay by using the Proteasome Activity Assay Kit (ab107921; Abcam) according to the manufacturer's protocol. The chymotrypsin-like activity of proteasome was measured by the cleavage of a synthetic proteasome substrate linked to methyl coumarin amide (MCA). Cleavage of MCA in the presence or absence of the proteasome inhibitor MG132 was detected by fluorescence emission using a Synergy^TM^2 Multi-Mode Microplate Reader (BioTek). Proteasome activity (U/ml) was calculated by subtracting for each sample, the background fluorescence given by the presence of the proteasome inhibitor MG132 (except in Supplementary Figure S7H) and by using an MCA standard curve.

### siRNA transfection

Cells were transfected with siRNA duplexes using Dharmafect 4 transfection reagent (GE Healthcare) for 24 h before inducing quiescence for 72 h. siRNAs used are directed against cullin 3 (Silencer^®^ Pre-designed siRNA CUL3; ID#217187; Ambion), cullin 4B (Silencer^®^ Pre-designed siRNA CUL4B; ID#13299; Ambion), DNA-PK (M-005030–01; GE Healthcare), TDP1 (M-016112–01; GE Healthcare), XLF (5′-CGCUGAUUCGAGAUCGAUUGAdTdT-3′; Eurogentec), XRCC4 (5′-AUAUGUUGGUGAACUGAGAdTdT-3′; Qiagen) or a nontargeting sequence (SR-CL000–005; Eurogentec).

### Statistics

Unless indicated otherwise, experimental differences were tested for significance using one-way ANOVA Tukey's multiple comparisons test with GraphPad Prism 6 software.

## RESULTS

### Top1cc stabilization induces transcription-dependent DSBs in quiescent cells

To investigate the production and signaling of transcription-dependent DSBs, we used primary human WI38 fibroblasts immortalized with hTERT (40). Non-transformed cells normally have low genomic instability (46) and can be induced in quiescence following serum deprivation (47,48), thus allowing the analysis of replication-independent damage produced by stabilized Top1cc.

Microscopy analysis of bromodeoxyuridine (BrdU) incorporation showed that the percentage of WI38 hTERT cells in S-phase decreased from 26% to approximately 3% after a 3-day serum deprivation (0.2% serum) (Supplementary Figure S1A and B), indicating that they efficiently entered quiescence. To determine whether CPT can induce replication-independent DSBs in those cells, we examined the phosphorylated histone H2AX on S139 (known as γH2AX) and its accumulation in nuclear foci. A single γH2AX focus reflects hundreds to thousands of γH2AX proteins that are concentrated around at least one DSB (49). CPT induced γH2AX foci within 1 h, with an average of 2 to 10 foci per nucleus at CPT concentrations ranging from 1 to 25 μM, respectively (Supplementary Figure S1C–E). Almost all cells formed at least 2 γH2AX foci at concentrations ≥ 10 μM (Supplementary Figure S1C and D). Similar results were obtained in serum-starved quiescent human IMR90 primary lung fibroblasts (Supplementary Figure S2A–D) and in serum-starved quiescent NHDF (Supplementary Figure S2E–H). These γH2AX foci colocalized with 53BP1, another DDR protein (Supplementary Figure S3A), and a neutral Comet assay provided direct evidence for the presence of DSBs (Supplementary Figure S3C and D). These results indicate that CPT induces replication-independent DSBs in quiescent cells.

To determine whether these breaks depend on transcription, we used the transcription inhibitor flavopiridol (FLV). Supplementary Figure S3A and B shows that FLV suppressed γH2AX and 53BP1 foci in CPT-treated quiescent WI38 hTERT cells. To test whether FLV prevented DSBs or prevented DDR signaling by some other mechanisms, we performed neutral Comet assays. The significant decrease in Comet tail moment in quiescent cells co-treated with FLV and CPT provides direct evidence for DSB suppression (Supplementary Figure S3C and D). Together, these results indicate that CPT induces transcription-dependent DSBs in serum-starved quiescent cells, providing a robust model to further study their production and their signaling.

### The formation of co-transcriptional DSBs requires Top1 proteolysis

**Figure 1.**
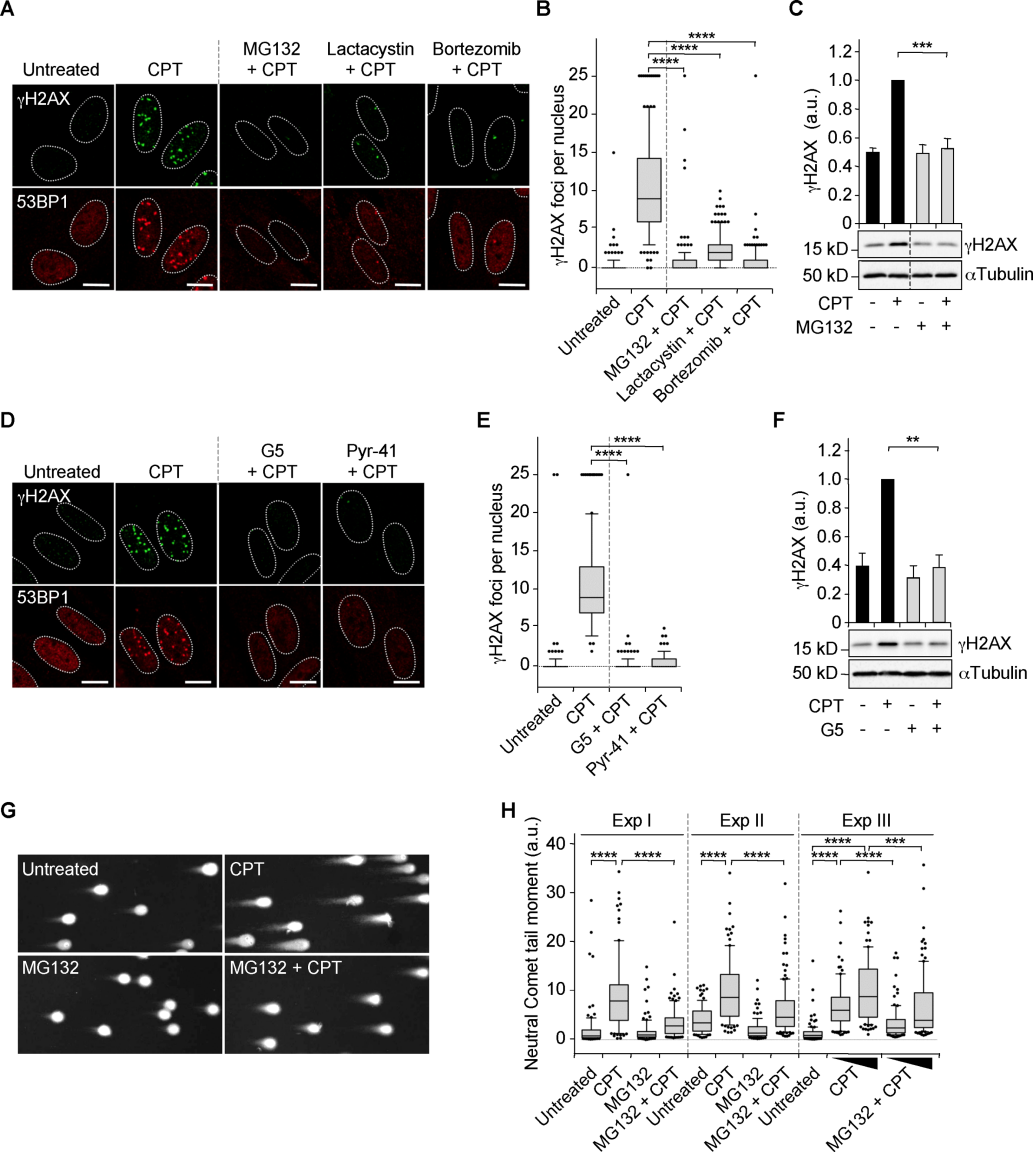
Induction of ubiquitin/proteasome-dependent DSBs in CPT-treated quiescent WI38 hTERT cells. (**A–F**) Serum-starved cells were treated with DMSO (1 h) or with MG132 (50 μM, 1 h), lactacystin (10 μM, 1 h), bortezomib (1 μM, 4 h), G5 (1.5 μM, 0.5 h) or Pyr-41 (9 μM, 0.5 h) before the addition of DMSO (untreated) or 25 μM CPT for 1 h and then co-stained for γH2AX (green) and 53BP1 (red) or analyzed by Western blot. ‘-’ in panels C and F means cells treated with DMSO. (**A** and **D**) Representative pictures. Nuclear contours, identified by DAPI staining (not shown), are indicated by dashed lines. Bars: 10 μm. (**B** and **E**) Number of γH2AX foci per nucleus from two independent experiments (147–153 nuclei were analyzed for each treatment). ****P < 0.0001. (**C** and **F**) Western blot of γH2AX. αTubulin: loading control. Dashed lines indicate that intervening wells have been spliced out. The top panels show quantification of γH2AX normalized to αTubulin (means ± SEM, n = 4 in panel C, n = 3 in panel F). ***P < 0.001; **P < 0.01. (**G** and **H**) Detection of DSBs by neutral Comet assays in serum-starved cells treated with DMSO or MG132 (25 μM) for 1 h before the addition of DMSO (untreated) or CPT for 1 h (7.5 μM for experiments (Exp) I and II; 5 and 7.5 μM for Exp III). (**G**) Representative pictures of nuclei from Exp I. (**H**) Quantification of neutral Comet tail moments for three independent experiments (95–133 nuclei were analyzed for each treatment in each experiment). ***P < 0.001; ****P < 0.0001. The untreated and CPT data from Exp I are from the same experiment as that of Supplementary Figure S3D.

Top1cc-mediated transcription block triggers Top1 degradation by the ubiquitin/proteasome system (12,13,50). Hence, we examined whether Top1 degradation would contribute to the formation of transcription-dependent DSBs. As expected, inhibition of transcription (FLV), ubiquitin (isopeptidase inhibitor G5) or proteasome (MG132) prevented Top1 degradation in CPT-treated quiescent WI38 hTERT cells (Supplementary Figure S4A–C). Then, we assessed whether inhibiting the ubiquitin/proteasome system would prevent DDR signaling. The proteasome inhibitors MG132, lactacystin and bortezomib (also called PS-341) (Figure 1A and B) as well as the ubiquitin inhibitors G5 and Pyr-41 (Figure 1D and E) prevented the induction of γH2AX and 53BP1 foci. Western blot analysis confirmed the suppressive effect of MG132 and G5 on γH2AX (Figure 1C and F). Importantly, DSB formation was also reduced by MG132, as indicated by a neutral Comet assay (Figure 1G and H). These results indicate that the ubiquitin/proteasome system is required for the formation of DSBs in CPT-treated quiescent cells.

**Figure 2.**
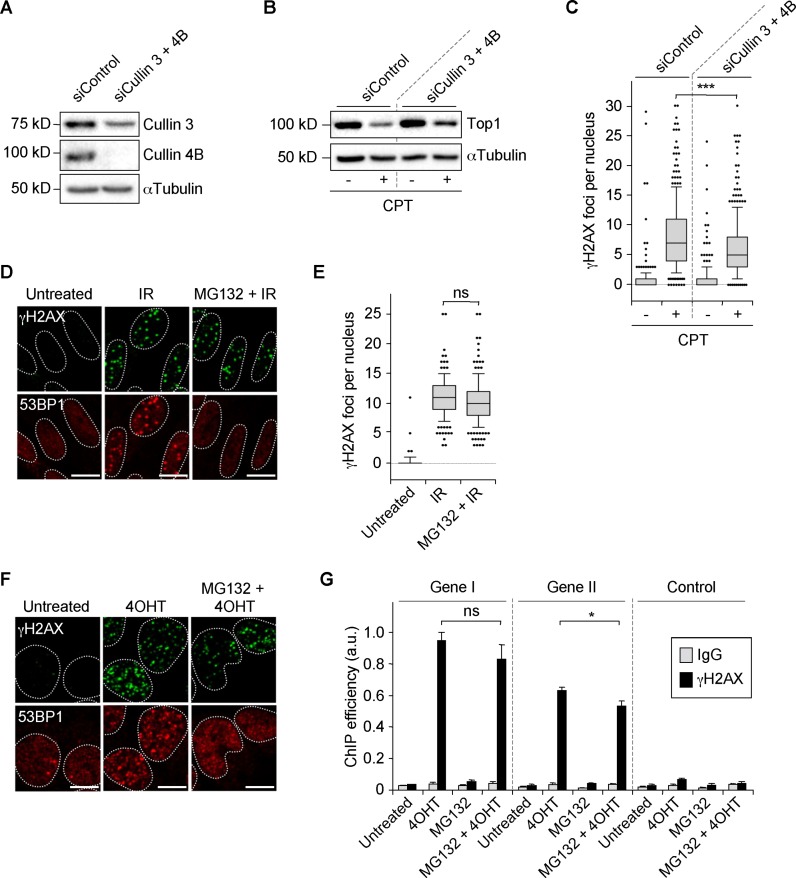
The production of DSBs depends on Top1 degradation in CPT-treated quiescent cells. (**A–C**) Serum-starved WI38 hTERT cells were co-transfected with siRNAs against cullin 3 and cullin 4B or against a control sequence and then treated with DMSO (−CPT) or 25 μM CPT (+CPT) for 1 h. (**A** and **B**) Western blotting of the indicated proteins. αTubulin: loading control. (**C**) Number of γH2AX foci per nucleus from one representative experiment (246–348 nuclei were analyzed for each treatment) out of three. ***P < 0.001. (**D** and **E**) Serum-starved WI38 hTERT cells were treated with DMSO or MG132 (50 μM) for 1 h before exposure to 0.8 Gy IR. One hour post-irradiation, cells were co-stained for γH2AX (green) and 53BP1 (red). (**D**) Representative pictures. (**E**) Number of γH2AX foci per nucleus from one representative experiment (162–180 nuclei were analyzed for each treatment) out of three. Ns: not significant. (**F** and **G**) U2OS EV28 cells were treated with DMSO or MG132 (10 μM) for 1 h before the addition of ethanol (untreated) or 300 nM 4-hydroxitamoxifen (4OHT) for 4 h to express *Asi*SI in the nucleus (42). (**F**) Representative pictures of cells co-stained for γH2AX (green) and 53BP1 (red). (**G**) ChIP analysis using an anti-γH2AX antibody (black) or a non-immune antibody (IgG, gray). Enrichment was assessed by QPCR amplification using primers proximal to two *Asi*SI sites located inside two genes (Gene I: SFRS6, Gene II: CCD47) and primers distal to an *Asi*SI site (Control). Enrichment was normalized to the maximum recovery for each experiment (means ± SEM, n = 3). Ns: not significant; *P < 0.05. In the microscopic images, nuclear contours, identified by DAPI staining (not shown), are indicated by dashed lines. Bars: 10 μm.

To evaluate whether defective Top1 degradation accounts for the lack of DSBs following inhibition of the ubiquitin/proteasome system, we inhibited cullin 3 and cullin 4B, which targets Top1 for proteosomal degradation in CPT-treated cells (43,51). siRNA-mediated depletion of cullin 3 and cullin 4B in quiescent WI38 hTERT cells (Figure 2A) reduced CPT-induced Top1 degradation (Figure 2B) and γH2AX foci (Figure 2C). To further assess the role of Top1 degradation, we induced DSBs with ionizing radiation (IR) and the restriction enzyme *Asi*SI (42), which do not trigger Top1 degradation in quiescent WI38 hTERT cells an in cycling U2OS cells, respectively (Supplementary Figure S4D and E). Under these conditions, MG132 did not suppress γH2AX foci induced by IR (Figure 2D and E) and by *Asi*SI (Figure 2F) indicating that genotoxics that do not promote Top1 degradation produce proteasome-independent DSBs. As expected, MG132, which causes depletion of free nuclear ubiquitin (52), prevented the ubiquitin-dependent focal accumulation of 53BP1 at DSB sites (31) in response to IR (Figure 2D) and *Asi*SI (Figure 2F). IR and *Asi*SI produce DSBs in both transcribed and non-transcribed regions whereas CPT likely produces co-transcriptional DSBs solely in transcribed regions. To assess whether proteasome activity is required simply because DSBs are located at transcribed regions, we analyzed γH2AX by ChIP at *Asi*SI sites located in genes. Figure 2G shows that MG132 did not suppress the induction of γH2AX in the two genes analyzed. These results suggest that the requirement of proteasome activity for CPT-induced co-transcriptional DSBs is not only because these breaks are produced at transcribed regions but also because Top1cc are stabilized on chromatin. Altogether, these findings strongly suggest that the ubiquitin/proteasome-dependent degradation of Top1 is required for the production of transcription-dependent DSBs following CPT treatment.

### Co-transcriptional DSBs arise from SSB intermediates generated after Top1 proteolysis and before TDP1 action

Top1 degradation primes the repair of transcription-blocking Top1cc by TDP1. Top1cc excision by TDP1 requires prior proteolysis of Top1 to expose the covalent bond between the Top1 catalytic tyrosine and the 3′-end of the DNA to be attacked. TDP1 generates a 3′-phosphate, which is hydrolyzed by polynucleotide kinase 3′-phosphatase (PNKP) before religation by ligase III (see Supplementary Figure S4F) (2,9–11,14). Because TDP1 deficient cells accumulate Top1 peptide-linked SSBs and causes hypersensitivity to CPTs (10,53,54), we examined whether transcription-dependent DSBs would arise from these SSB intermediates generated before TDP1 action.

**Figure 3.**
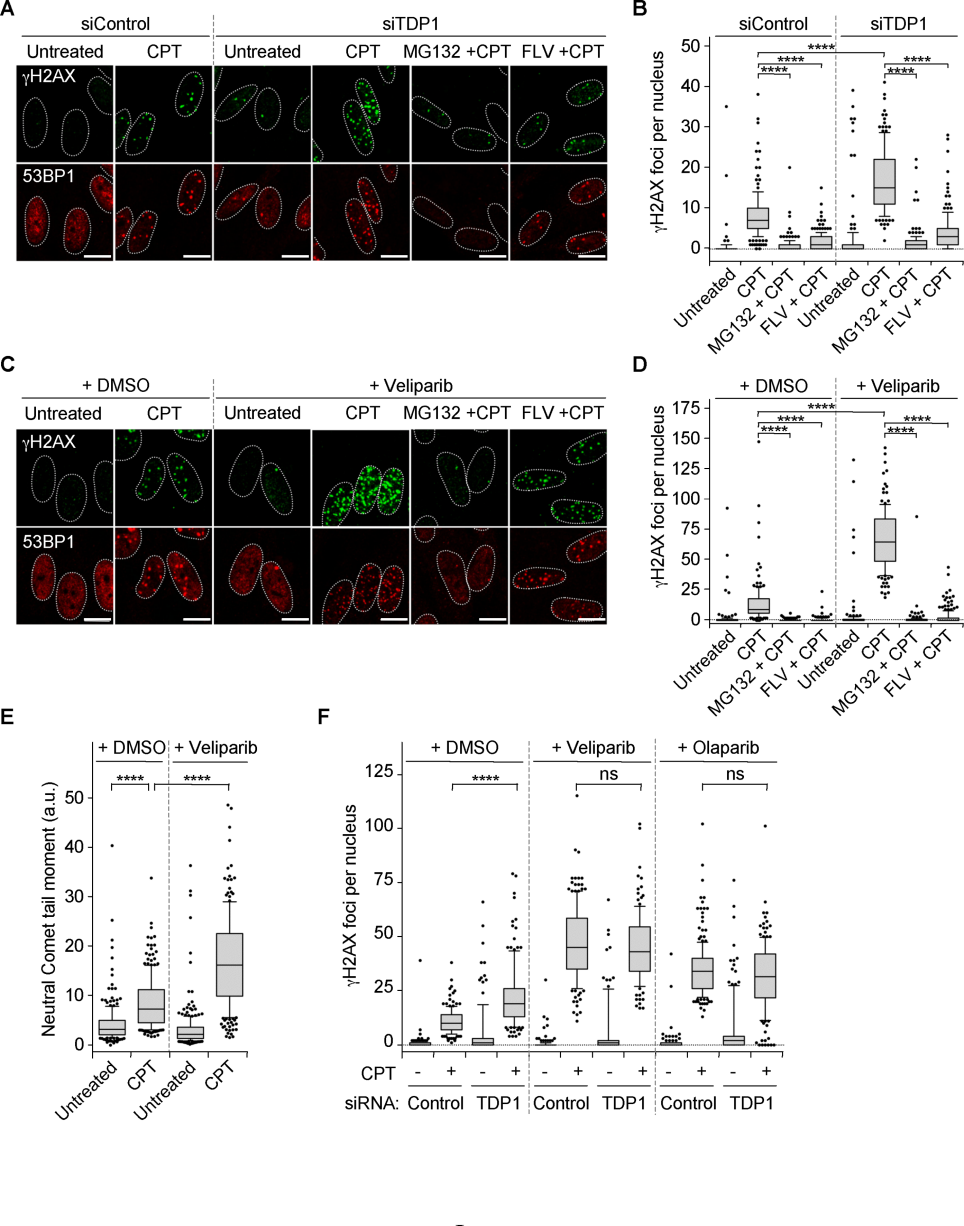
Inhibition of TDP1 or PARP increases CPT-induced DSBs in quiescent WI38 hTERT cells. (**A** and **B**) Serum-starved cells were transfected with TDP1-targeting or non-targeting (Control) siRNAs and then treated with DMSO or with MG312 (25 μM) or FLV (1 μM) for 1 h before the addition of DMSO (untreated) or CPT (25 μM) for 1 h. Cells were then co-stained for γH2AX (green) and 53BP1 (red). (**A**) Representative pictures. (**B**) Number of γH2AX foci per nucleus from one representative experiment (152–233 nuclei were analyzed for each treatment) out of two to four. ****P < 0.0001. (**C** and **D**) Serum-starved cells were treated for 1 h with DMSO or veliparib (5 μM) in combination with DMSO or with MG132 (25 μM) or FLV (1 μM), before the addition of DMSO (untreated) or CPT (25 μM) for 1 h. Cells were then co-stained for γH2AX (green) and 53BP1 (red). (**C**) Representative pictures. (**D**) Number of γH2AX foci per nucleus from one representative experiment (120–223 nuclei were analyzed for each treatment) out of two. ****P < 0.0001. (**E**) Detection of DSBs by neutral Comet assays in serum-starved cells treated with DMSO or veliparib (5 μM) before the addition of DMSO (untreated) or CPT (7.5 μM). Data show the quantification of one representative experiment (226–249 nuclei were analyzed for each treatment) out of three. ****P < 0.0001. (**F**) Serum-starved cells were transfected with TDP1-targeting or non-targeting (Control) siRNAs and then treated for 1 h with DMSO or with veliparib (5 μM) or olaparib (10 μM) before the addition of DMSO (−CPT) or 25 μM CPT (+CPT) for 1 h. Cells were then stained for γH2AX. The number of γH2AX foci per nucleus from one representative experiment (105–215 nuclei were analyzed for each treatment) out of two is shown. ****P < 0.0001; ns, not significant. In the microscopic images, nuclear contours, identified by DAPI staining (not shown), are indicated by dashed lines. Bars: 10 μm.

Depletion of TDP1 with siRNA markedly increased the number of γH2AX and 53BP1 foci in CPT-treated quiescent WI38 hTERT cells (Figure 3A and B and Supplementary Figure S4G). Previous work showed that Top1cc excision by TDP1 requires PARP1 (11,55). PARP1 binds to and PARylates TDP1 leading to TDP1 stabilization and its recruitment at Top1cc-induced DNA damage sites. We therefore assessed whether inhibition of PARP1 would also increase DSBs in CPT-treated quiescent cells. The PARP inhibitors veliparib (also called ABT-888) and olaparib (also called AZD-2281), both increased the number of γH2AX and 53BP1 foci (Figure 3C and D and Supplementary Figure S4H and I). DSB formation was also increased by veliparib, as indicated by a neutral Comet assay (Figure 3E). Inhibition of transcription (FLV) or proteasome (MG132), which prevented CPT-induced Top1 degradation (Supplementary Figure S4A and C), also prevented CPT-induced accumulation of γH2AX and 53BP1 foci following TDP1 depletion (Figure 3A and B) and PARP inhibition (Figure 3C and D), indicating that those breaks depend on Top1 degradation. To determine whether TDP1 and PARP1 are in the same pathway to prevent DSBs, we compared the number of γH2AX foci following co-treatment with CPT and PARP inhibitors when TDP1 is expressed or not. Figure 3F shows that TDP1 suppression with siRNA did not further increase the number of γH2AX foci in quiescent WI38 hTERT cells exposed to CPT and veliparib or CPT and olaparib, indicating that TDP1 and PARP1 are in the same pathway. Together, these results indicate that co-transcriptional DSBs produced in CPT-treated quiescent cells arise from SSB intermediates generated after Top1 proteolysis and before TDP1 action.

### Activation of ATM and DNA-PK by co-transcriptional DSBs

**Figure 4.**
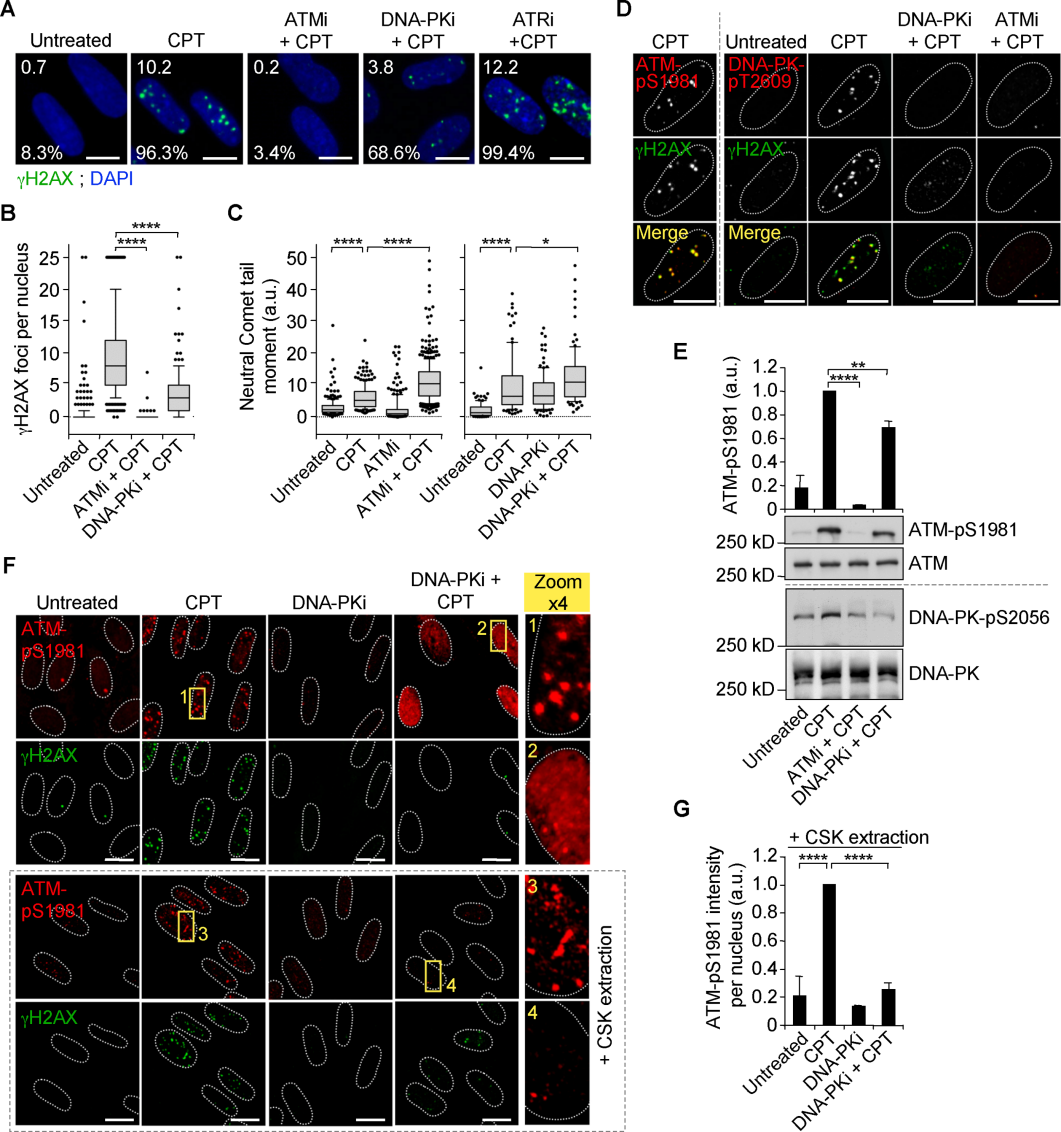
Inhibition of DNA-PK prevents the formation of ATM-pS1981 foci in CPT-treated quiescent WI38 hTERT cells. (**A** and **B**) Serum-starved cells were treated with DMSO or with ATMi (10 μM), DNA-PKi (10 μM) or ATRi (10 μM) for 1 h before the addition of DMSO (untreated) or CPT (25 μM) for 1 h. Cells were then stained for γH2AX (green) and DNA was counterstained with DAPI (blue). (**A**) Representative pictures. Numbers are the percentage of nuclei with at least 2 γH2AX foci (bottom) and the average number of γH2AX foci per nucleus (top). (**B**) Number of γH2AX foci per nucleus. Quantifications in panels A and B are from one representative experiment (159–306 nuclei were analyzed for each treatment) out of three. ****P < 0.0001. (**C**) Detection of DSBs by neutral Comet assays in serum-starved cells treated with DMSO or with ATMi (10 μM) or DNA-PKi (10 μM) for 1 h before the addition of DMSO (untreated) or CPT (7.5 μM) for 1 h. Data show the quantification of one representative experiment out of two for ATMi (226–428 nuclei were analyzed for each treatment) and out of four for DNA-PKi (102–120 nuclei were analyzed for each treatment). ****P < 0.0001; *P < 0.05. The untreated and CPT data from the experiment with ATMi are from the same experiment as that of Figure 3E. (**D**) Left panels: colocalization of ATM-pS1981 foci (red) with γH2AX foci (green) in serum-starved cells treated with CPT (25 μM) for 1 h before staining. Similar data were obtained in >10 independent experiments. Right panels: serum-starved cells were treated with DMSO or with DNA-PKi (10 μM) or ATMi (10 μM) for 1 h before the addition of DMSO (untreated) or CPT (25 μM) for 1 h and then co-stained for DNA-PK-pT2609 (red) and γH2AX (green). Similar data were obtained in two independent experiments. Images were merged to determine colocalization (yellow). (**E**) Western blot of ATM-pS1981 and DNA-PK-pS2056 in serum-starved cells treated as described for panel D. The top panel shows quantification of ATM-pS1981 (means ± SD, n = 3). **P < 0.01; ****P < 0.0001. (**F**) Serum-starved cells were treated with DMSO or DNA-PKi (10 μM) for 1 h before the addition of DMSO (untreated) or CPT (25 μM) for 1 h. Nucleosoluble proteins were then removed (lower panels) or not (top panels) with CSK buffer before co-staining for ATM-pS1981 (red) and γH2AX (green). Representative images are shown. The zoomed images are x4 magnifications of the main images. The yellow boxes indicate areas of magnification. (**G**) Quantification of ATM-pS1981 fluorescence intensity per nucleus shown in lower panel F (means ± SD, n = 3, 70–100 nuclei were analyzed for each treatment in each experiment). ****P < 0.0001. In the microscopic images, nuclear contours, identified by DAPI staining (not shown), are indicated by dashed lines. Bars: 10 μm.

Next, we studied the signaling of these co-transcriptional DSBs to gain insight into their functional relevance. To that end, we examined which kinases phosphorylate H2AX. ATM, ATR and DNA-PK are the main kinases for H2AX in response to DNA damage (49). To identify which of them induces γH2AX, we assessed whether γH2AX foci formation is prevented by specific chemical inhibitors of these kinases in CPT-treated quiescent WI38 hTERT cells; the ATM inhibitor (ATMi) KU55933, the DNA-PK inhibitor (DNA-PKi) NU7441 and the ATR inhibitor (ATRi) VE-821. ATMi completely suppressed γH2AX foci and DNA-PKi markedly reduced their number and size (Figure 4A and B and Supplementary Figure S5A and B). Analysis of DSBs by neutral Comet assays showed that ATMi and DNA-PKi did not decrease the Comet tail moment induced by CPT (Figure 4C), which exclude the possibility that fewer DSBs account for the reduced γH2AX levels. Similar results were obtained in quiescent IMR90 cells (Supplementary Figure S2A–D) and in quiescent NHDF cells (Supplementary Figure S2E–H). By contrast, ATRi did not reduce the number of γH2AX foci induced by CPT in quiescent WI38 hTERT cells (Figure 4A) under conditions where it prevented phosphorylation of the ATR substrate Chk1 in replicating cells (Supplementary Figure S5C). This is consistent with ATR being primarily activated by replication stress (56) and that it is expressed at low levels in non-cycling cells (Supplementary Figure S5D) (17,57). These data suggest that ATM and DNA-PK but not ATR are implicated in the induction of γH2AX in CPT-treated quiescent cells.

We therefore assessed whether ATM and DNA-PK are activated under conditions where γH2AX is induced. ATM autophosphorylation on S1981 (ATM–pS1981) marks activated ATM (58). In quiescent WI38 hTERT cells, CPT induced ATM–pS1981 as previously reported (59) that colocalized with γH2AX foci (Figure 4D and Supplementary Figure S5E). The ATM targets KAP1, Chk2 and p53 (23) were also phosphorylated (Supplementary Figure S5E). Similar to ATM, DNA-PK phosphorylation status regulates its activity. DNA-PK autophosphorylation on both S2056 and T2609 is required for the repair of DSBs by NHEJ (60,61). Figure 4D and E shows that CPT induced DNA-PK phosphorylation on both residues in quiescent WI38 hTERT cells. Immunofluorescence microscopy further revealed that DNA-PK phosphorylated on T2609 formed discreet nuclear foci that colocalized with γH2AX (Figure 4D), indicating that similar to ATM, DNA-PK is activated at DSB sites. Previous work showed that ATM can phosphorylate DNA-PK on T2609 following IR (62). Inhibition of ATM prevented CPT-induced DNA-PK phosphorylation on T2609 and also on S2056 in quiescent WI38 hTERT cells (Figure 4D and E). Together, these data suggest that in CPT-treated quiescent cells, transcription-dependent DSBs activate ATM, which in turn activates DNA-PK.

### DNA-PK promotes the assembly of activated ATM into nuclear foci

Next, we considered whether cross talk between ATM and DNA-PK is mutual or limited to one direction in which ATM activates DNA-PK. To test this, we asked whether inhibiting DNA-PK would prevent MDC1 and 53BP1 foci, which depends, at least in part, on ATM (23). Supplementary Figure S5F shows that DNA-PKi suppressed CPT-induced MDC1 and 53BP1 foci in quiescent WI38 hTERT cells. To test more directly the role of DNA-PK on ATM, we analyzed the impact of DNA-PK inhibition on the induction of ATM-pS1981 and on its accumulation in nuclear foci. DNA-PKi only slightly reduced CPT-induced ATM-pS1981 levels (Figure 4E), indicating that DNA-PK does not markedly contribute to ATM activation. Microscopy analysis confirmed that DNA-PKi did not suppress ATM-pS1981 following CPT treatment (Figure 4F, top panels). However, it revealed a pan staining for ATM–pS1981 rather than well-defined foci (Figure 4F, top panels), suggesting that activated ATM is not localized at damaged sites. Previous work showed that ATM associated with sites of DSBs was resistant to detergent extraction while unbound ATM was removed (63). Thus, we treated cells with a detergent-based extraction buffer known as cytosqueleton (CSK) buffer (64) before immunofluorescence staining for ATM–pS1981. CSK buffer suppressed the pan-staining signal for ATM–pS1981 in cells co-treated with DNA-PKi and CPT under conditions where it did not affect ATM–pS1981 foci in cells treated with CPT alone (Figure 4F bottom panels and G). Together, these data suggest that DNA-PK promotes the assembly of activated ATM into nuclear foci in CPT-treated quiescent cells.

To assess whether the role of DNA-PK on ATM foci formation is related to its function in DSB repair, we inhibited XLF and XRCC4, which mediate DNA-PK-dependent NHEJ (33). Contrary to DNA-PK inhibition, depletion of XLF or XRCC4 with siRNAs did not prevent CPT-induced ATM-pS1981 foci in quiescent WI38 hTERT cells (Supplementary Figure S6). These results suggest that the role of DNA-PK on ATM foci formation is independent of its function in DSB repair.

### DNA-PK promotes ubiquitination of H2AX and H2A at DSB sites

H2AX and H2A are ubiquitinated in response to DSBs (27–32). Previous work showed that defective monoubiquitination of H2AX at K119/K120 in IR-exposed cells impaired the recruitment of ATM–pS1981 to DSBs and thereby reduced γH2AX and MDC1 foci formation (25,26), effects similar to those observed following DNA-PK inhibition in CPT-treated quiescent cells (Figure 4 and Supplementary Figure S5F). We therefore tested whether DNA-PK inhibition would prevent CPT-induced H2AX monoubiquitination in quiescent cells.

**Figure 5.**
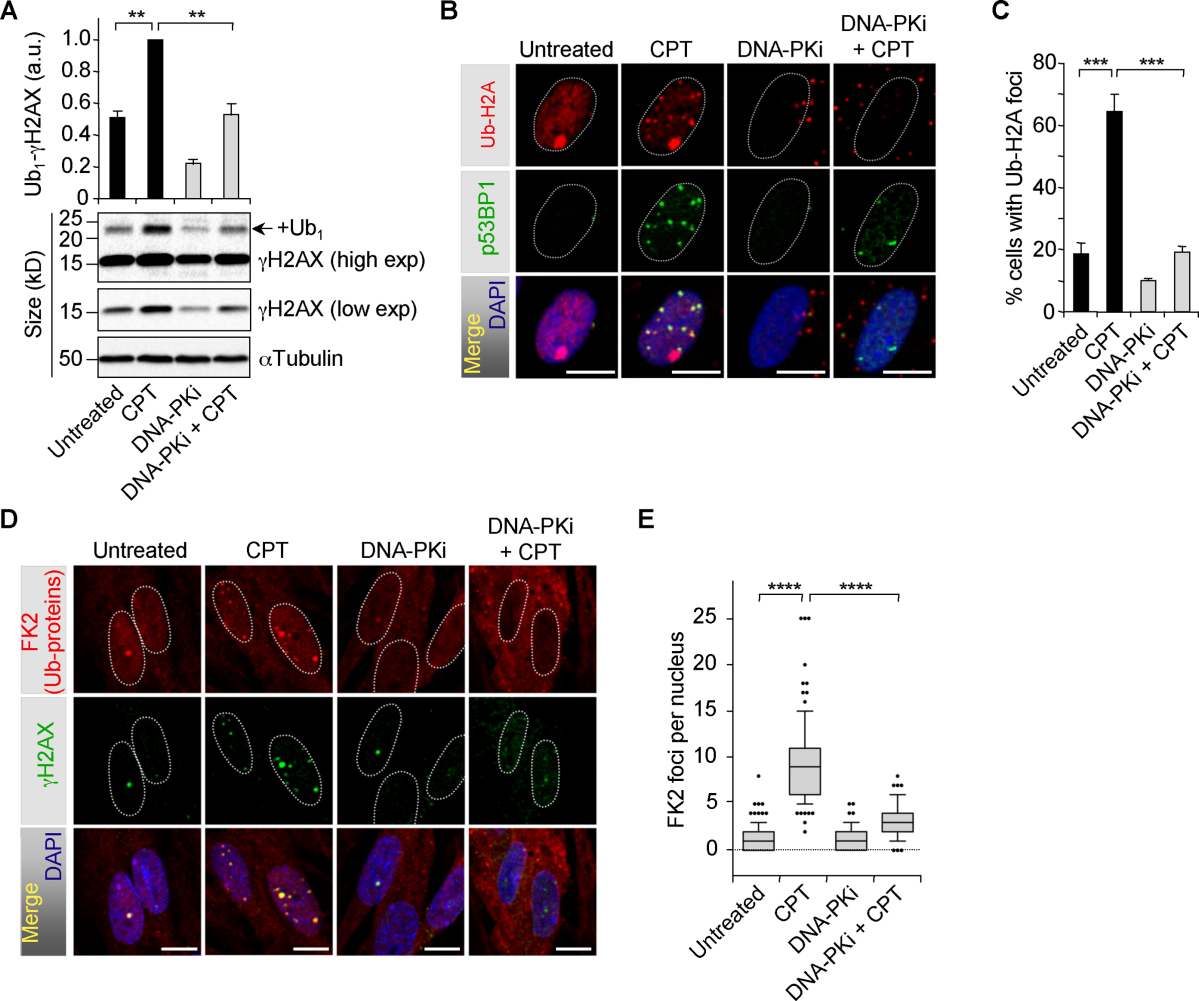
Inhibition of DNA-PK prevents monoubiquitination of H2AX and H2A in CPT-treated quiescent WI38 hTERT cells. (**A–E**) Serum-starved cells were treated with DMSO or DNA-PKi (10 μM) for 1 h before the addition of DMSO (untreated) or CPT (25 μM) for 1 h. (**A**) Western blot of γH2AX. +Ub1 indicates γH2AX monoubiquitinated. The top panel shows quantification of Ub1-γH2AX normalized to αTubulin (means ± SEM, n = 4). **P < 0.01. (**B** and **C**) Cells were pre-extracted with CSK buffer before co-staining for Ub-H2A (red) and 53BP1 phosphorylated on S1778 (p53BP1) (green). (**B**) Representative pictures. Images were merged to determine colocalization (yellow). The large Ub-H2AX focus at the periphery of the nuclei of untreated and CPT-treated cells may marks the inactive X chromosome as reported (91). (**C**) Percentages of nuclei with at least 5 Ub-H2A foci (means ± SEM, n = 3, 100 nuclei were analyzed for each treatment in each experiment). ***P < 0.001. (**D** and **E**) Cells were co-stained for ubiquitinated proteins (FK2, red) and γH2AX (green). (**D**) Representative pictures. Images were merged to determine colocalization (yellow). (**E**) Number of FK2 foci per nucleus from one representative experiment (76–111 nuclei were analyzed for each treatment) out of three. ****P < 0.0001. In the microscopic images, nuclear contours, identified by DAPI staining (blue in the merge images at bottom), are indicated by dashed lines. Bars: 10 μm.

Western blot analysis using an antibody against H2AX showed that CPT did not significantly increase global H2AX monoubiquitination in quiescent WI38 hTERT cells (Supplementary Figure S7A). It might be because H2AX is monoubiquitinated only at damaged sites in response to DSBs (26), and CPT induced only few γH2AX foci in quiescent cells (Supplementary Figure S1). Consistent with this hypothesis, detection of H2AX monoubiquitination by Western blot was previously reported at high doses of IR, typically 4 to 10 Gy (26,65). To examine specifically H2AX monoubiquitination at DSB sites, we analyzed γH2AX monoubiquitination. CPT induced γH2AX monoubiquitination in quiescent WI38 hTERT cells, which was prevented by DNA-PKi (Figure 5A). To analyze more directly histone monoubiquitination at DSB sites, immunofluorescence microscopy was performed with an antibody against H2A monoubiquitinated at K119 (Ub-H2A). CPT induced Ub-H2A foci that colocalized with the p53BP1 foci, and those Ub-H2A foci were completely prevented by DNA-PKi (Figure 5B and C). Consistent with these results, CPT also induced DNA-PK-dependent FK2 foci (Figure 5D and E), which mark ubiquitinated proteins at DNA damage sites (66). siRNA-mediated depletion of DNA-PK confirmed the suppressive effect of DNA-PKi on FK2 foci formation (Supplementary Figure S7B and C). Altogether, these results indicate that DNA-PK promotes monoubiquitination of H2AX and H2A at site of co-transcriptional DSBs in CPT-treated quiescent cells. This in turn may favor the recruitment of activated ATM and phosphorylation of its downstream substrates.

### DNA-PK promotes ubiquitin/proteasome-dependent Top1cc removal

**Figure 6.**
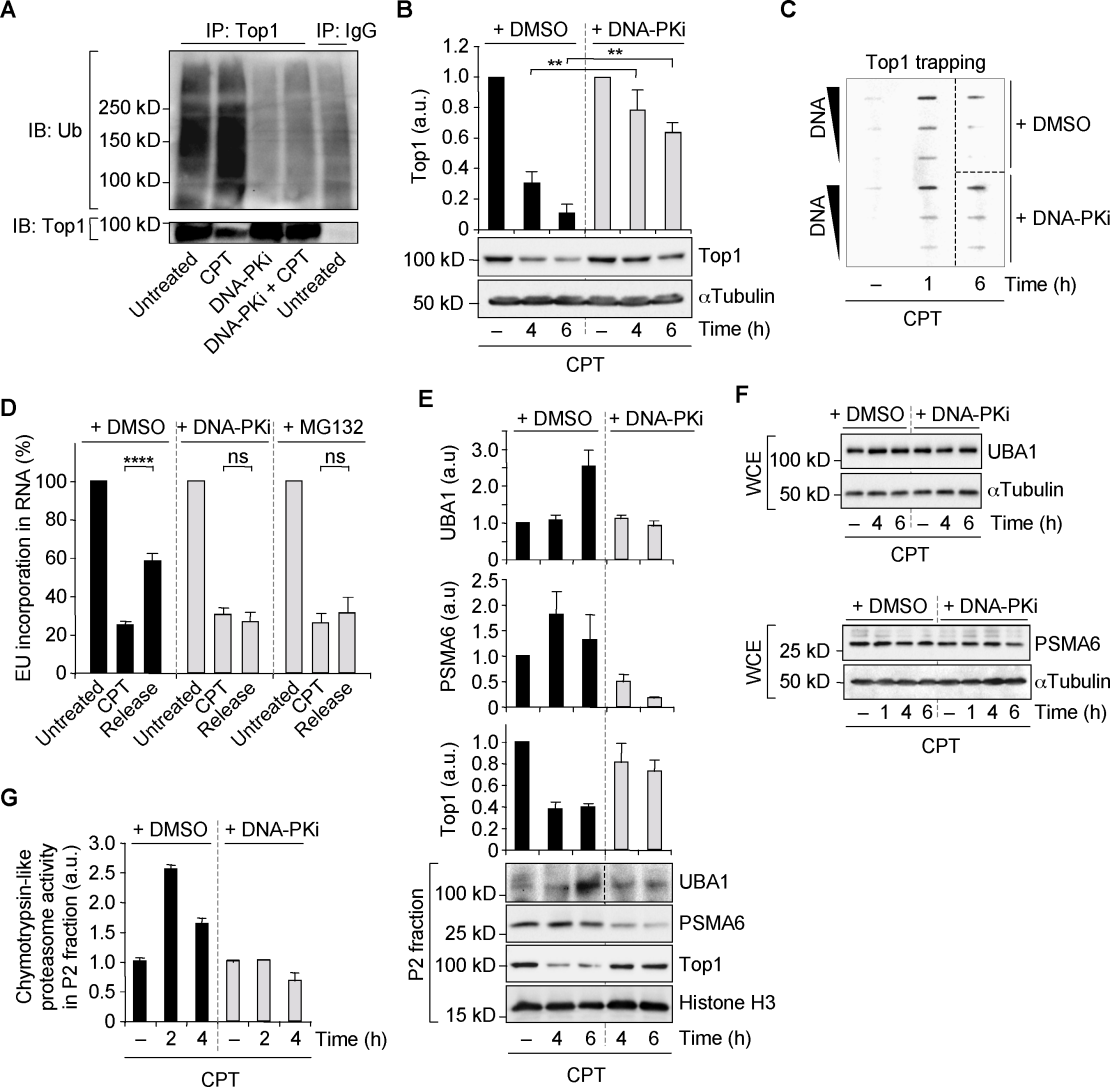
Inhibition of DNA-PK prevents Top1 degradation and proteasome activity in CPT-treated quiescent WI38 hTERT cells. (**A**) Serum-starved cells were treated with DMSO or DNA-PKi (10 μM) for 1 h before the addition of DMSO (untreated) or CPT (25 μM) for 1 h. Whole cell extracts were immunoprecipitated (IP) with Top1 or non immune (IgG) antibodies, and immunoblotted with ubiquitin or Top1 antibodies. (**B** and **C**) Serum-starved cells were treated with DMSO or DNA-PKi (10 μM) for 1 h before the addition of DMSO (− CPT) for 6 h or CPT (25 μM) for the indicated times. (**B**) Western blot of Top1 in whole cell extracts allowing the detection of the whole cellular Top1 (Top1 and Top1cc) as previously reported (13). The top panel shows quantification of Top1 normalized to αTubulin (means ± SEM, n = 4). **P < 0.01. (**C**) Detection of Top1-DNA cleavage complexes (Top1cc). Three concentrations of genomic DNA (5, 2.5 and 1.25 μg) were probed with an anti-Top1 antibody. Dashed lines indicate where panels have been reorganized to facilitate reading. (**D**) Serum-starved cells were treated with DMSO, DNA-PKi or MG132 for 1 h (untreated) before the addition of 25 μM CPT for 1 h (CPT). Cells were then washed 4 times in CPT-free medium containing DMSO, DNA-PKi or MG132 and cultured in CPT-free medium containing DMSO, DNA-PKi or MG132 for 3 h to allow for transcription recovery (release). For the last 30 min of each time point, cells were incubated with 1 mM 5-ethylnyl uridine (EU) to label newly synthesized RNA. DNA-PKi: 1 μM; MG132: 25 μM. Percentages of EU incorporation per nucleus were assessed by fluorescence microscopy and normalized to the level of untreated cells, which was taken at 100% (means ± SEM, n = 3, 126–326 nuclei were analyzed for each treatment in each experiment). ****P < 0.0001; ns: not significant. (**E–G**) Serum-starved cells were treated with DMSO or DNA-PKi (10 μM) for 1 h before the addition of DMSO (− CPT: 6 h in panels E,F and 4 h in panel G) or CPT (25 μM) for the indicated times. (**E**) Western blot of UBA1, PSMA6 and Top1 in the P2 fraction (see Supplementary Figure S7G). Dashed lines in the UBA1 blot indicate that intervening wells have been spliced out. The top panel shows quantification of UBA1, PSMA6 and Top1 in the P2 fraction normalized to histone H3 (mean ± SEM; n = 4 for PSMA6 and Top1, n = 3 for UBA1). (**F**) Western blot of UBA1 (top) and PSMA6 (bottom) in whole cell extracts (WCE). (**G**) Chymotrypsin-like activity assessment in the P2 fraction (means ± SD of triplicates; each time point is representative of at least two independent experiments). αTubulin and histone H3 were the loading controls for Western blot experiments.

Based on our results that DNA-PK promotes ubiquitination processes (Figure 5) and the fact that transcription-blocking Top1cc are removed following ubiquitination and proteosomal degradation of Top1 (Supplementary Figure S4A–C) (12,13,50), we examined the possibility that DNA-PK could promote Top1 ubiquitination and degradation. Immunoprecipitation of Top1 followed by immunoblotting of ubiquitin showed that DNA-PKi prevented CPT-induced Top1 ubiquitination in quiescent WI38 hTERT cells (Figure 6A). Consistent with this, DNA-PKi also markedly decreased CPT-induced Top1 degradation in quiescent WI38 hTERT cells (Figure 6B) as well as in quiescent IMR90 and NHDF cells (Supplementary Figure S7D). Analysis of endogenous Top1cc confirmed that defective Top1 degradation following DNA-PK inhibition reflected defective Top1cc removal (Figure 6C). Because defective Top1cc removal prevents resumption of RNA synthesis (9,12,67), we assessed whether DNA-PK inhibition would prevent transcription recovery following CPT removal. In quiescent WI38 hTERT cells, approximately 50% of transcription levels were recovered 3 h after termination of the CPT treatment (Figure 6D). Under these conditions, transcription levels were not significantly recovered in the presence of DNA-PKi (Figure 6D). As a control, the proteasome inhibitor MG132, which prevented Top1 degradation (Supplementary Figure S4C), also prevented transcription recovery (Figure 6D) as previously reported (12). The role of DNA-PK in promoting Top1 degradation is unlikely related to its function in NHEJ repair as depletion of XRCC4 or XPF with siRNAs did not affect CPT-induced Top1 degradation in quiescent WI38 hTERT cells (Supplementary Figure S7E). Because ATM activates DNA-PK (Figure 4D and E), we examined whether ATM inhibition would also prevent Top1 degradation. Supplementary Figure S7F shows that ATMi also decreased CPT-induced Top1 degradation in quiescent WI38 hTERT cells.

Consistent with the ubiquitin/proteasome-dependent removal of Top1cc, the E1 ubiquitin ligase UBA1 and the 20S-proteasome subunit PSMA6, which are both involved in DDR (68,69), tended to accumulate in chromatin-bound fraction (P2 fraction, see Supplementary Figure S7G) in CPT-treated quiescent WI38 hTERT cells (Figure 6E). Under these conditions, the global expression of PSMA6 remained unchanged while that of UBA1 slightly increased (Figure 6F). Accumulation of UBA1 and PSMA6 in P2 fraction was further associated with an increased activity of proteasome (Figure 6G). DNA-PKi suppressed the CPT-induced UBA1 and PSMA6 accumulation and proteasome activity in P2 fraction (Figure 6E–G). As a control, DNA-PKi did not directly inhibit the proteasome (Supplementary Figure S7H). Altogether, these results suggest that DNA-PK promotes ubiquitin/proteasome-dependent removal of Top1cc in CPT-treated quiescent cells.

### Transcription- and proteasome-dependent apoptosis of quiescent cells by stabilized Top1cc

**Figure 7.**
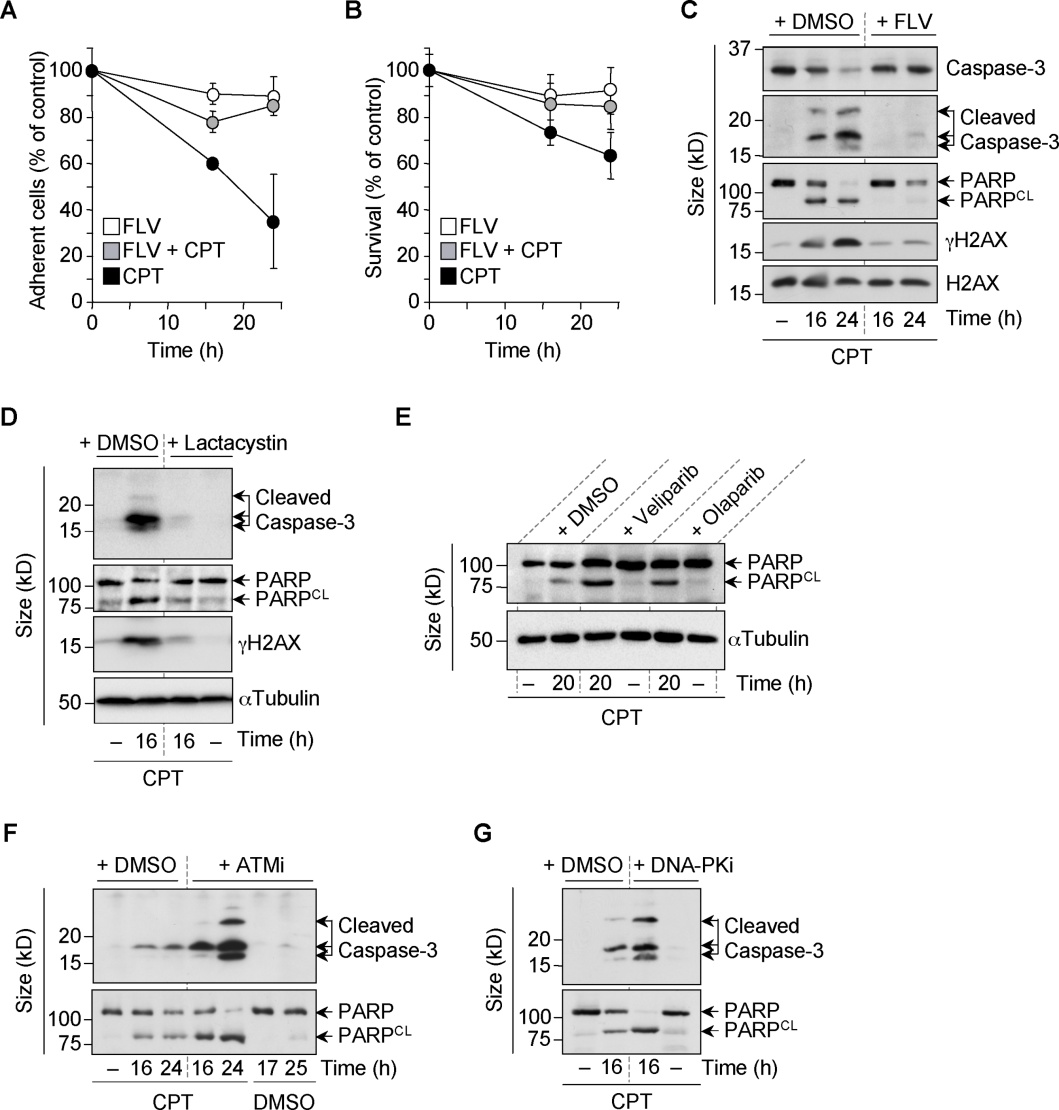
CPT induces transcription- and proteasome-dependent apoptosis in quiescent WI38 hTERT cells. (**A** and **B**) Serum-starved cells were treated with DMSO or FLV (1 μM) for 1 h before the addition of DMSO or CPT (25 μM) for 16 and 24 h. (**A**) Percentages of cells that remained attached to culture flasks (means ± SD of quadruplicates). (**B**) Cell survival was analyzed by a CellTiter-Blue assay (means ± SD of triplicates). (**C–G**) Western blot of the indicated proteins in serum-starved cells treated for 1 h with DMSO or with FLV (1 μM) (**C**), lactacystin (10 μM) (**D**), veliparib (5 μM) or olaparib (10 μM) (**E**), ATMi (10 μM) (**F**) or DNA-PKi (10 μM) (**G**) before the addition of DMSO (‘-’, 24 h in panels C and F) or CPT (25 μM) for the indicated times. Data shown are representatives from two to three experiments. PARP^CL^: cleaved PARP. H2AX and αTubulin were the loading controls.

To assess whether transcription-dependent DSBs can kill non-replicative cells, we analyzed the percentage of quiescent cells that remained attached to the culture flask following CPT treatment. Typically, cells detach as they undergo apoptosis. CPT decreased the number of quiescent WI38 hTERT cells attached to the culture flask by approximately 70% after 24 h (Figure 7A). It also reduced cell viability assessed by a CellTiter-Blue assay (Figure 7B). This was associated with the appearance of biochemical markers of apoptosis such as the cleavage of caspase-3 and PARP, and the massive induction of γH2AX, which was reported in cells undergoing apoptosis (70) (Figure 7C). All these apoptotic marks were suppressed by the transcription inhibitor FLV (Figure 7A–C), suggesting that transcription-dependent DSBs are the initiating events for apoptosis induction. Consistent with this hypothesis, inhibition of CPT-induced DSBs with lactacystin (Figure 1A and B) also prevented caspase-3 and PARP cleavage and γH2AX induction (Figure 7D). By contrast, increased CPT-induced DSBs with veliparib or olaparib (Figure 3C–F and Supplementary Figure S4H and I) also increased PARP cleavage (Figure 7E).

To analyze the impact of DDR signaling on CPT-induced apoptosis of quiescent cells, we inhibited ATM and DNA-PK. Figure 7F and G shows that ATMi and DNA-PKi, both increased CPT-induced caspase-3 and PARP cleavage, indicating that the predominant role of ATM- and DNA-PK-dependent signaling is likely to promote cell survival after CPT-induced transcription-dependent DSBs.

## DISCUSSION

**Figure 8.**
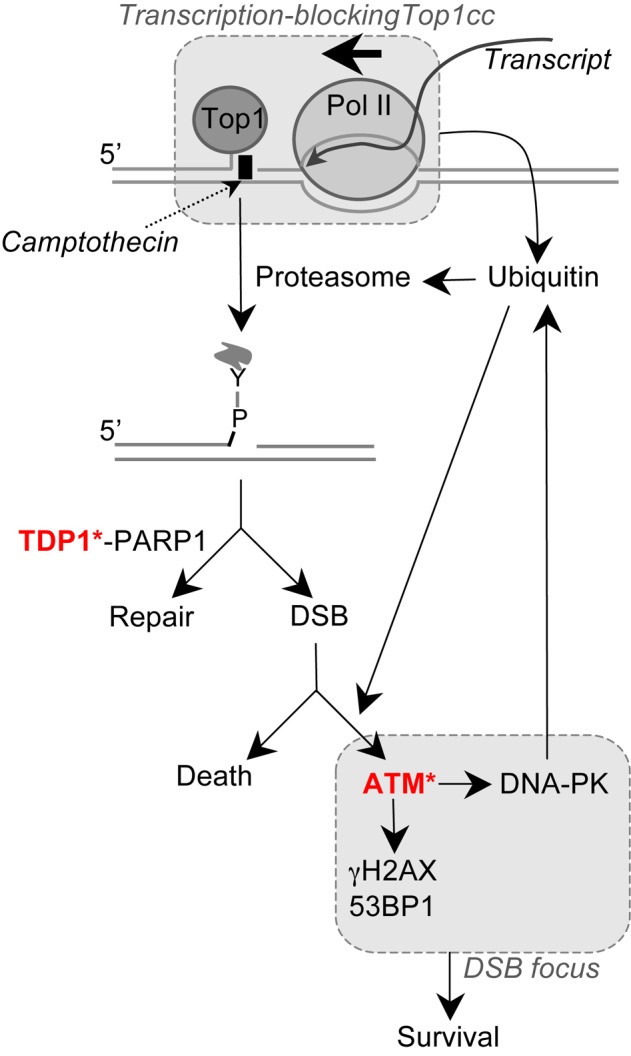
Proposed molecular pathways for the production and signaling of DSBs by transcription-blocking Top1 lesions. ‘Y’ is the catalytic tyrosine of Top1 covalently bound to the 3′-end of the broken DNA. Asterisks indicate proteins whose deficiency induces neurodegenerative diseases (TDP1 deficiency: SCAN1 syndrome; ATM deficiency: AT syndrome).

Although defective repair of DSBs can lead to neurodegenerative diseases, the molecular processes of their production and signaling in non-replicating cells are largely unknown. Here, we analyzed the transcription-dependent DSBs that form in non-replicating cells as a consequence of Top1cc stabilization (17). Our data support a model depicted in Figure 8 in which Top1cc stabilization blocks transcription elongation, which triggers partial Top1 proteolysis and the generation of a Top1 peptide-linked SSB, which is a substrate for TDP1. Defective repair of this SSB intermediate by TDP1 can give rise to a DSB, which leads to ATM activation and phosphorylation of its substrates such as H2AX and 53BP1. ATM also activates DNA-PK, which promotes H2AX and H2A monoubiquitination and the assembly of activated ATM into nuclear foci. ATM and DNA-PK also increase Top1 proteolysis suggesting that this pathway further enhances Top1cc repair after DSB induction. This is consistent with recent work showing that ATM deficiency increases Top1cc levels in CPT-treated quiescent astrocytes (71,72). Because we found that Top1 proteolysis primes DSB formation, DNA-PK could therefore increases DSB production in a feedback loop. This might be however compensated as DNA-PK can also phosphorylate TDP1, which increases its repair activity toward Top1cc (73). Nevertheless, we did not find that DNA-PK inhibition reduced the amount of DSBs in CPT-treated quiescent cells measured by a neutral Comet assay. DSBs event tend to accumulate, which might be related to the function of DNA-PK that we report here in promoting ATM signaling but also on its function in NHEJ repair (33). It would be interesting to determine whether this model in serum-starved quiescent cells could be extended to neuronal cells.

Transcription-blocking Top1cc seem primarily repaired by the TDP1 excision pathway. Indeed, our study and previous reports indicate that Top1 degradation is transcription dependent following CPT exposure (12,13,50) and that TDP1 primarily repairs transcription-blocking Top1cc as compared to replication-blocking Top1cc (9,10). Consistent with the involvement of the TDP1 pathway in the repair of transcription-blocking Top1cc, Top1 degradation has been suggested to promote resumption of RNA synthesis (12). Our findings that DSBs produced in CPT-treated quiescent cells depend both on transcription and on Top1 degradation suggest that they arise during the repair of Top1cc. A further support to this is that inhibition of TDP1 or PARP1 (which is required for TDP1 activity (11,55)), increases transcription-dependent DSBs following Top1cc stabilization. Because TDP1 deficient cells accumulate Top1 peptide-linked SSB intermediates (9,10,53,54), our results suggest that transcription-dependent DSBs arise from these intermediates if TDP1 fails to repair them.

How a Top1 peptide-linked SSB can give rise to a DSB in non-replicating cells is still an unresolved question. The presence of a nearby SSB on opposing DNA strand would result in a DSB. Different non-exclusive mechanisms can be envisaged for the production of this second SSB. First, it might result from the repair of another trapped Top1cc, which is plausible considering that Top1 tends to be concentrated in transcribed regions (1,74). The repair of this trapped Top1cc might involve the TDP1 excision pathway that would generate another Top1 peptide-linked SSB or the endonuclease pathway (XPF, Mre11, SLX4, CtIP) that would generate a single-strand DNA gap (2,11). Although two nearby Top1cc on opposing DNA strands might happen simply by chance, it is possible that it is more controlled, e.g. that they form at genomic loci with convergent transcription units. Second, a SSB on opposing DNA strand might result from the repair of an endogenous DNA lesion that would generate a single-strand DNA nick or gap (75). Lastly, a SSB might result from the cleavage of an R-loop, possibly by transcription-coupled nucleotide excision (TC-NER) factors (21). Indeed, transcription-blocking Top1 lesions can cause R-loop formation as a result of negative supercoiling behind transcription complexes (76), inhibition of the SR-kinase activity of Top1 (77–79) and/or chromatin displacement of spliceosomes (80), which have been implicated in the formation of transcription-dependent DSBs in post-mitotic cells (17). Analysis of the genomic distribution of DSBs, Top1cc and R-loops in CPT-treated quiescent cells is likely to provide new insights into the mechanism of DSB production by transcription-blocking Top1 lesions.

It is now well documented that DNA-PK functions in cellular processes other than NHEJ such as gene regulation (81,82) and mitosis (83–85). Here, we report a novel function of DNA-PK in the regulation of protein ubiquitination. Previous work suggested connection between DNA-PK and the proteasome. In the presence of DSBs, DNA-PK was reported to induce transcription arrest by a mechanism that depends on proteasome activity (86). Also, DNA-PK inhibition prevents the accumulation of the proteasome activator PA200 on chromatin in response to IR (87). Phosphorylation is known to regulate protein ubiquitination in two main ways (88). First, substrate phosphorylation can create a recognition signal for binding of an E3 ligase. Second, phosphorylation of an E3 ligase can stimulate its ubiquitin transfer activity. Hence, it is possible that DNA-PK phosphorylates H2AX, H2A and Top1 and/or their respective E3 ligases following CPT treatment. The first possibility is plausible as the N-terminal domain of Top1 binds to DNA-PK (89) and possesses a potential phosphorylation site for DNA-PK (SQ motif on S10). DNA-PK can also phosphorylate H2AX on S139 (γH2AX) (34) but it is unlikely that this is what primes H2AX for ubiquitination because H2A lacks S139 but is also ubiquitinated in response to DSBs. Several E3 ubiquitin ligases have been reported for H2AX and H2A (RNF2, RNF8, RNF168) (25–31) and for Top1 (Cullin3, Cullin4A, Cullin4B, BRCA1) (13,43,51) but it is not known whether they can be phosphorylated by DNA-PK. It is also possible that DNA-PK favors the recruitment of the E1 ligase UBA1 at damaged sites as we found that DNA-PK inhibition prevented CPT-induced UBA1 accumulation on chromatin.

Lastly, our analysis demonstrates that co-transcriptional DSBs can kill non-replicating cells following CPT treatment. Co-transcriptional DSBs might occur spontaneously in cells as Top1cc can be trapped under a broad range of physiological conditions including oxidative base damages, alkylation by carcinogenic compounds and nicks (see Table 1 in reference (2)), and by ribonucleotide misincorporation (3–5). Therefore, these co-transcriptional DSBs may have a marked impact on non-replicating cell fate. Neurodegenerative diseases can arise from defective repair of Top1cc (SCAN1 caused by TDP1 deficiency) (16) as well as from defective response to DSBs (AT caused by ATM deficiency) (24). Neurons might be particularly prone to produce co-transcriptional DSBs as a result of high rates of oxygen consumption, which produces reactive oxygen species that can stabilize Top1cc (1,2,90). Hence, our findings raise the possibility that accumulation of co-transcriptional DSBs and the defective response to those breaks might contribute to the neurodegenerative phenotype of SCAN1 and AT patients, respectively.

## Supplementary Material

SUPPLEMENTARY DATA

## References

[1] Pommier Y. (2006). Topoisomerase I inhibitors: camptothecins and beyond. Nat. Rev. Cancer.

[2] Pommier Y., Huang S.Y., Gao R., Das B.B., Murai J., Marchand C. (2014). Tyrosyl-DNA-phosphodiesterases (TDP1 and TDP2). DNA Rep..

[3] Huang S.Y., Ghosh S., Pommier Y. (2015). Topoisomerase I alone is sufficient to produce short DNA deletions and can also reverse nicks at ribonucleotide sites. J. Biol. Chem..

[4] Kim N., Huang S.Y., Williams J.S., Li Y.C., Clark A.B., Cho J.E., Kunkel T.A., Pommier Y., Jinks-Robertson S. (2011). Mutagenic processing of ribonucleotides in DNA by yeast topoisomerase I. Science.

[5] Sparks J.L., Burgers P.M. (2015). Error-free and mutagenic processing of topoisomerase 1-provoked damage at genomic ribonucleotides. EMBO J..

[6] Capranico G., Ferri F., Fogli M.V., Russo A., Lotito L., Baranello L. (2007). The effects of camptothecin on RNA polymerase II transcription: roles of DNA topoisomerase I. Biochimie.

[7] Ljungman M., Lane D.P. (2004). Transcription - guarding the genome by sensing DNA damage. Nat. Rev. Cancer.

[8] Pouliot J.J., Yao K.C., Robertson C.A., Nash H.A. (1999). Yeast gene for a Tyr-DNA phosphodiesterase that repairs topoisomerase I complexes. Science.

[9] El-Khamisy S.F., Saifi G.M., Weinfeld M., Johansson F., Helleday T., Lupski J.R., Caldecott K.W. (2005). Defective DNA single-strand break repair in spinocerebellar ataxia with axonal neuropathy-1. Nature.

[10] Miao Z.H., Agama K., Sordet O., Povirk L., Kohn K.W., Pommier Y. (2006). Hereditary ataxia SCAN1 cells are defective for the repair of transcription-dependent topoisomerase I cleavage complexes. DNA Rep..

[11] Pommier Y., Barcelo J.M., Rao V.A., Sordet O., Jobson A.G., Thibaut L., Miao Z.H., Seiler J.A., Zhang H., Marchand C. (2006). Repair of topoisomerase I-mediated DNA damage. Prog. Nucleic Acid Res. Mol. Biol..

[12] Desai S.D., Zhang H., Rodriguez-Bauman A., Yang J.M., Wu X., Gounder M.K., Rubin E.H., Liu L.F. (2003). Transcription-dependent degradation of topoisomerase I-DNA covalent complexes. Mol. Cell Biol..

[13] Sordet O., Larochelle S., Nicolas E., Stevens E.V., Zhang C., Shokat K.M., Fisher R.P., Pommier Y. (2008). Hyperphosphorylation of RNA polymerase II in response to topoisomerase I cleavage complexes and its association with transcription- and BRCA1-dependent degradation of topoisomerase I. J. Mol. Biol..

[14] Ashour M.E., Atteya R., El-Khamisy S.F. (2015). Topoisomerase-mediated chromosomal break repair: an emerging player in many games. Nat. Rev. Cancer.

[15] Rass U., Ahel I., West S.C. (2007). Defective DNA repair and neurodegenerative disease. Cell.

[16] Takashima H., Boerkoel C.F., John J., Saifi G.M., Salih M.A., Armstrong D., Mao Y., Quiocho F.A., Roa B.B., Nakagawa M. (2002). Mutation of TDP1, encoding a topoisomerase I-dependent DNA damage repair enzyme, in spinocerebellar ataxia with axonal neuropathy. Nat. Genet..

[17] Sordet O., Redon C.E., Guirouilh-Barbat J., Smith S., Solier S., Douarre C., Conti C., Nakamura A.J., Das B.B., Nicolas E. (2009). Ataxia telangiectasia mutated activation by transcription- and topoisomerase I-induced DNA double-strand breaks. EMBO Rep..

[18] Sordet O., Nakamura A.J., Redon C.E., Pommier Y. (2010). DNA double-strand breaks and ATM activation by transcription-blocking DNA lesions. Cell Cycle.

[19] Zhang Y.W., Regairaz M., Seiler J.A., Agama K.K., Doroshow J.H., Pommier Y. (2011). Poly(ADP-ribose) polymerase and XPF-ERCC1 participate in distinct pathways for the repair of topoisomerase I-induced DNA damage in mammalian cells. Nucleic Acids Res..

[20] Aguilera A., Garcia-Muse T. (2012). R loops: from transcription byproducts to threats to genome stability. Mol. Cell.

[21] Sollier J., Stork C.T., Garcia-Rubio M.L., Paulsen R.D., Aguilera A., Cimprich K.A. (2014). Transcription-coupled nucleotide excision repair factors promote R-loop-induced genome instability. Mol. Cell.

[22] Lukas J., Lukas C., Bartek J. (2011). More than just a focus: the chromatin response to DNA damage and its role in genome integrity maintenance. Nat. Cell Biol..

[23] Shiloh Y., Ziv Y. (2013). The ATM protein kinase: regulating the cellular response to genotoxic stress, and more. Nat. Rev..

[24] Savitsky K., Bar-Shira A., Gilad S., Rotman G., Ziv Y., Vanagaite L., Tagle D.A., Smith S., Uziel T., Sfez S. (1995). A single ataxia telangiectasia gene with a product similar to PI-3 kinase. Science.

[25] Facchino S., Abdouh M., Chatoo W., Bernier G. (2010). BMI1 confers radioresistance to normal and cancerous neural stem cells through recruitment of the DNA damage response machinery. J. Neurosci..

[26] Pan M.R., Peng G., Hung W.C., Lin S.Y. (2011). Monoubiquitination of H2AX protein regulates DNA damage response signaling. J. Biol. Chem..

[27] Doil C., Mailand N., Bekker-Jensen S., Menard P., Larsen D.H., Pepperkok R., Ellenberg J., Panier S., Durocher D., Bartek J. (2009). RNF168 binds and amplifies ubiquitin conjugates on damaged chromosomes to allow accumulation of repair proteins. Cell.

[28] Stewart G.S., Panier S., Townsend K., Al-Hakim A.K., Kolas N.K., Miller E.S., Nakada S., Ylanko J., Olivarius S., Mendez M. (2009). The RIDDLE syndrome protein mediates a ubiquitin-dependent signaling cascade at sites of DNA damage. Cell.

[29] Huen M.S., Grant R., Manke I., Minn K., Yu X., Yaffe M.B., Chen J. (2007). RNF8 transduces the DNA-damage signal via histone ubiquitylation and checkpoint protein assembly. Cell.

[30] Kolas N.K., Chapman J.R., Nakada S., Ylanko J., Chahwan R., Sweeney F.D., Panier S., Mendez M., Wildenhain J., Thomson T.M. (2007). Orchestration of the DNA-damage response by the RNF8 ubiquitin ligase. Science.

[31] Mailand N., Bekker-Jensen S., Faustrup H., Melander F., Bartek J., Lukas C., Lukas J. (2007). RNF8 ubiquitylates histones at DNA double-strand breaks and promotes assembly of repair proteins. Cell.

[32] Mattiroli F., Vissers J.H., van Dijk W.J., Ikpa P., Citterio E., Vermeulen W., Marteijn J.A., Sixma T.K. (2012). RNF168 ubiquitinates K13–15 on H2A/H2AX to drive DNA damage signaling. Cell.

[33] Davis A.J., Chen B.P., Chen D.J. (2014). DNA-PK: a dynamic enzyme in a versatile DSB repair pathway. DNA Rep..

[34] Stiff T., O'Driscoll M., Rief N., Iwabuchi K., Lobrich M., Jeggo P.A. (2004). ATM and DNA-PK function redundantly to phosphorylate H2AX after exposure to ionizing radiation. Cancer Res..

[35] Yang Y., Kitagaki J., Dai R.M., Tsai Y.C., Lorick K.L., Ludwig R.L., Pierre S.A., Jensen J.P., Davydov I.V., Oberoi P. (2007). Inhibitors of ubiquitin-activating enzyme (E1), a new class of potential cancer therapeutics. Cancer Res..

[36] Aleo E., Henderson C.J., Fontanini A., Solazzo B., Brancolini C. (2006). Identification of new compounds that trigger apoptosome-independent caspase activation and apoptosis. Cancer Res..

[37] Hickson I., Zhao Y., Richardson C.J., Green S.J., Martin N.M., Orr A.I., Reaper P.M., Jackson S.P., Curtin N.J., Smith G.C. (2004). Identification and characterization of a novel and specific inhibitor of the ataxia-telangiectasia mutated kinase ATM. Cancer Res..

[38] Reaper P.M., Griffiths M.R., Long J.M., Charrier J.D., Maccormick S., Charlton P.A., Golec J.M., Pollard J.R. (2011). Selective killing of ATM- or p53-deficient cancer cells through inhibition of ATR. Nat. Chem. Biol..

[39] Leahy J.J., Golding B.T., Griffin R.J., Hardcastle I.R., Richardson C., Rigoreau L., Smith G.C. (2004). Identification of a highly potent and selective DNA-dependent protein kinase (DNA-PK) inhibitor (NU7441) by screening of chromenone libraries. Bioorg. Med. Chem. Lett..

[40] Jeanblanc M., Ragu S., Gey C., Contrepois K., Courbeyrette R., Thuret J.Y., Mann C. (2012). Parallel pathways in RAF-induced senescence and conditions for its reversion. Oncogene.

[41] Aasen T., Izpisua Belmonte J.C. (2010). Isolation and cultivation of human keratinocytes from skin or plucked hair for the generation of induced pluripotent stem cells. Nat. Protoc..

[42] Iacovoni J.S., Caron P., Lassadi I., Nicolas E., Massip L., Trouche D., Legube G. (2010). High-resolution profiling of gammaH2AX around DNA double strand breaks in the mammalian genome. EMBO J..

[43] Kerzendorfer C., Whibley A., Carpenter G., Outwin E., Chiang S.C., Turner G., Schwartz C., El-Khamisy S., Raymond F.L., O'Driscoll M. (2010). Mutations in Cullin 4B result in a human syndrome associated with increased camptothecin-induced topoisomerase I-dependent DNA breaks. Hum. Mol. Genet..

[44] Drouet J., Delteil C., Lefrancois J., Concannon P., Salles B., Calsou P. (2005). DNA-dependent protein kinase and XRCC4-DNA ligase IV mobilization in the cell in response to DNA double strand breaks. J. Biol. Chem..

[45] Regairaz M., Zhang Y.W., Fu H., Agama K.K., Tata N., Agrawal S., Aladjem M.I., Pommier Y. (2011). Mus81-mediated DNA cleavage resolves replication forks stalled by topoisomerase I-DNA complexes. J. Cell Biol..

[46] Lobrich M., Shibata A., Beucher A., Fisher A., Ensminger M., Goodarzi A.A., Barton O., Jeggo P.A. (2010). gammaH2AX foci analysis for monitoring DNA double-strand break repair: strengths, limitations and optimization. Cell Cycle.

[47] Dimri G.P., Hara E., Campisi J. (1994). Regulation of two E2F-related genes in presenescent and senescent human fibroblasts. J. Biol. Chem..

[48] Coller H.A., Sang L., Roberts J.M. (2006). A new description of cellular quiescence. PLoS Biol..

[49] Bonner W.M., Redon C.E., Dickey J.S., Nakamura A.J., Sedelnikova O.A., Solier S., Pommier Y. (2008). gammaH2AX and cancer. Nat. Rev. Cancer.

[50] Desai S.D., Li T.K., Rodriguez-Bauman A., Rubin E.H., Liu L.F. (2001). Ubiquitin/26S proteasome-mediated degradation of topoisomerase I as a resistance mechanism to camptothecin in tumor cells. Cancer Res..

[51] Zhang H.F., Tomida A., Koshimizu R., Ogiso Y., Lei S., Tsuruo T. (2004). Cullin 3 promotes proteasomal degradation of the topoisomerase I-DNA covalent complex. Cancer Res..

[52] Dantuma N.P., Groothuis T.A., Salomons F.A., Neefjes J. (2006). A dynamic ubiquitin equilibrium couples proteasomal activity to chromatin remodeling. J. Cell Biol..

[53] Katyal S., el-Khamisy S.F., Russell H.R., Li Y., Ju L., Caldecott K.W., McKinnon P.J. (2007). TDP1 facilitates chromosomal single-strand break repair in neurons and is neuroprotective in vivo. EMBO J..

[54] Interthal H., Chen H.J., Kehl-Fie T.E., Zotzmann J., Leppard J.B., Champoux J.J. (2005). SCAN1 mutant Tdp1 accumulates the enzyme–DNA intermediate and causes camptothecin hypersensitivity. EMBO J..

[55] Das B.B., Huang S.Y., Murai J., Rehman I., Ame J.C., Sengupta S., Das S.K., Majumdar P., Zhang H., Biard D. (2014). PARP1-TDP1 coupling for the repair of topoisomerase I-induced DNA damage. Nucleic Acids Res..

[56] Zeman M.K., Cimprich K.A. (2014). Causes and consequences of replication stress. Nat. Cell Biol..

[57] Jones G.G., Reaper P.M., Pettitt A.R., Sherrington P.D. (2004). The ATR-p53 pathway is suppressed in noncycling normal and malignant lymphocytes. Oncogene.

[58] Bakkenist C.J., Kastan M.B. (2003). DNA damage activates ATM through intermolecular autophosphorylation and dimer dissociation. Nature.

[59] Lin C.P., Ban Y., Lyu Y.L., Desai S.D., Liu L.F. (2008). A ubiquitin-proteasome pathway for the repair of topoisomerase I-DNA covalent complexes. J. Biol. Chem..

[60] Chan D.W., Chen B.P., Prithivirajsingh S., Kurimasa A., Story M.D., Qin J., Chen D.J. (2002). Autophosphorylation of the DNA-dependent protein kinase catalytic subunit is required for rejoining of DNA double-strand breaks. Genes Dev..

[61] Chen B.P., Chan D.W., Kobayashi J., Burma S., Asaithamby A., Morotomi-Yano K., Botvinick E., Qin J., Chen D.J. (2005). Cell cycle dependence of DNA-dependent protein kinase phosphorylation in response to DNA double strand breaks. J. Biol. Chem..

[62] Chen B.P., Uematsu N., Kobayashi J., Lerenthal Y., Krempler A., Yajima H., Lobrich M., Shiloh Y., Chen D.J. (2007). Ataxia telangiectasia mutated (ATM) is essential for DNA-PKcs phosphorylations at the Thr-2609 cluster upon DNA double strand break. J. Biol. Chem..

[63] Andegeko Y., Moyal L., Mittelman L., Tsarfaty I., Shiloh Y., Rotman G. (2001). Nuclear retention of ATM at sites of DNA double strand breaks. J. Biol. Chem..

[64] Rodriguez R., Miller K.M., Forment J.V., Bradshaw C.R., Nikan M., Britton S., Oelschlaegel T., Xhemalce B., Balasubramanian S., Jackson S.P. (2012). Small-molecule-induced DNA damage identifies alternative DNA structures in human genes. Nat. Chem. Biol..

[65] Mosammaparast N., Kim H., Laurent B., Zhao Y., Lim H.J., Majid M.C., Dango S., Luo Y., Hempel K., Sowa M.E. (2013). The histone demethylase LSD1/KDM1A promotes the DNA damage response. J. Cell Biol..

[66] Morris J.R., Solomon E. (2004). BRCA1 : BARD1 induces the formation of conjugated ubiquitin structures, dependent on K6 of ubiquitin, in cells during DNA replication and repair. Hum. Mol. Genet..

[67] Alagoz M., Wells O.S., El-Khamisy S.F. (2014). TDP1 deficiency sensitizes human cells to base damage via distinct topoisomerase I and PARP mechanisms with potential applications for cancer therapy. Nucleic Acids Res..

[68] Moudry P., Lukas C., Macurek L., Hanzlikova H., Hodny Z., Lukas J., Bartek J. (2012). Ubiquitin-activating enzyme UBA1 is required for cellular response to DNA damage. Cell cycle.

[69] Levy-Barda A., Lerenthal Y., Davis A.J., Chung Y.M., Essers J., Shao Z., van Vliet N., Chen D.J., Hu M.C., Kanaar R. (2011). Involvement of the nuclear proteasome activator PA28gamma in the cellular response to DNA double-strand breaks. Cell cycle.

[70] Solier S., Sordet O., Kohn K.W., Pommier Y. (2009). Death receptor-induced activation of the Chk2- and histone H2AX-associated DNA damage response pathways. Mol. Cell Biol..

[71] Alagoz M., Chiang S.C., Sharma A., El-Khamisy S.F. (2013). ATM deficiency results in accumulation of DNA-topoisomerase I covalent intermediates in neural cells. PLoS One.

[72] Katyal S., Lee Y., Nitiss K.C., Downing S.M., Li Y., Shimada M., Zhao J., Russell H.R., Petrini J.H., Nitiss J.L. (2014). Aberrant topoisomerase-1 DNA lesions are pathogenic in neurodegenerative genome instability syndromes. Nat. Neurosci..

[73] Das B.B., Antony S., Gupta S., Dexheimer T.S., Redon C.E., Garfield S., Shiloh Y., Pommier Y. (2009). Optimal function of the DNA repair enzyme TDP1 requires its phosphorylation by ATM and/or DNA-PK. EMBO J..

[74] Khobta A., Ferri F., Lotito L., Montecucco A., Rossi R., Capranico G. (2006). Early effects of topoisomerase I inhibition on RNA polymerase II along transcribed genes in human cells. J. Mol. Biol..

[75] Pourquier P., Pilon A.A., Kohlhagen G., Mazumder A., Sharma A., Pommier Y. (1997). Trapping of mammalian topoisomerase I and recombinations induced by damaged DNA containing nicks or gaps. Importance of DNA end phosphorylation and camptothecin effects. J. Biol. Chem..

[76] Drolet M., Broccoli S., Rallu F., Hraiky C., Fortin C., Masse E., Baaklini I. (2003). The problem of hypernegative supercoiling and R-loop formation in transcription. Front Biosci..

[77] Soret J., Gabut M., Dupon C., Kohlhagen G., Stevenin J., Pommier Y., Tazi J. (2003). Altered serine/arginine-rich protein phosphorylation and exonic enhancer-dependent splicing in Mammalian cells lacking topoisomerase I. Cancer Res..

[78] Tuduri S., Crabbe L., Conti C., Tourriere H., Holtgreve-Grez H., Jauch A., Pantesco V., De Vos J., Thomas A., Theillet C. (2009). Topoisomerase I suppresses genomic instability by preventing interference between replication and transcription. Nat. Cell Biol..

[79] Li X., Manley J.L. (2005). Inactivation of the SR protein splicing factor ASF/SF2 results in genomic instability. Cell.

[80] Tresini M., Warmerdam D.O., Kolovos P., Snijder L., Vrouwe M.G., Demmers J.A., van I.W.F., Grosveld F.G., Medema R.H., Hoeijmakers J.H. (2015). The core spliceosome as target and effector of non-canonical ATM signalling. Nature.

[81] Ju B.G., Lunyak V.V., Perissi V., Garcia-Bassets I., Rose D.W., Glass C.K., Rosenfeld M.G. (2006). A topoisomerase IIbeta-mediated dsDNA break required for regulated transcription. Science.

[82] Wong R.H., Chang I., Hudak C.S., Hyun S., Kwan H.Y., Sul H.S. (2009). A role of DNA-PK for the metabolic gene regulation in response to insulin. Cell.

[83] Lee K.J., Lin Y.F., Chou H.Y., Yajima H., Fattah K.R., Lee S.C., Chen B.P. (2011). Involvement of DNA-dependent protein kinase in normal cell cycle progression through mitosis. J. Biol. Chem..

[84] Shang Z.F., Huang B., Xu Q.Z., Zhang S.M., Fan R., Liu X.D., Wang Y., Zhou P.K. (2010). Inactivation of DNA-dependent protein kinase leads to spindle disruption and mitotic catastrophe with attenuated checkpoint protein 2 Phosphorylation in response to DNA damage. Cancer Res..

[85] Wang C.Y., Kao Y.H., Lai P.Y., Chen W.Y., Chung B.C. (2013). Steroidogenic factor 1 (NR5A1) maintains centrosome homeostasis in steroidogenic cells by restricting centrosomal DNA-dependent protein kinase activation. Mol. Cell Biol..

[86] Pankotai T., Bonhomme C., Chen D., Soutoglou E. (2012). DNAPKcs-dependent arrest of RNA polymerase II transcription in the presence of DNA breaks. Nat. Struct. Mol. Biol..

[87] Blickwedehl J., Agarwal M., Seong C., Pandita R.K., Melendy T., Sung P., Pandita T.K., Bangia N. (2008). Role for proteasome activator PA200 and postglutamyl proteasome activity in genomic stability. Proc. Natl. Acad. Sci. U.S.A..

[88] Hunter T. (2007). The age of crosstalk: phosphorylation, ubiquitination, and beyond. Mol. Cell.

[89] Czubaty A., Girstun A., Kowalska-Loth B., Trzcinska A.M., Purta E., Winczura A., Grajkowski W., Staron K. (2005). Proteomic analysis of complexes formed by human topoisomerase I. Biochim. Biophys. Acta.

[90] Daroui P., Desai S.D., Li T.K., Liu A.A., Liu L.F. (2004). Hydrogen peroxide induces topoisomerase I-mediated DNA damage and cell death. J. Biol. Chem..

[91] Fang J., Chen T., Chadwick B., Li E., Zhang Y. (2004). Ring1b-mediated H2A ubiquitination associates with inactive X chromosomes and is involved in initiation of X inactivation. J. Biol. Chem..

