# Supplementary material for: DNA-PK triggers histone ubiquitination and signaling in response to DNA double-strand breaks produced during the repair of transcription-blocking topoisomerase I lesions
Source: Nucleic Acids Res. 2015 Nov 17;44(3):1161–78. doi: 10.1093/nar/gkv1196 (PMC4756817; doi:10.1093/nar/gkv1196)
Supplement: SUPPLEMENTARY DATA [file supp_44_3_1161__index.html]

DNA-PK triggers histone ubiquitination and signaling in response to DNA double-strand breaks produced during the repair of transcription-blocking topoisomerase I lesions — SUPPLEMENTARY DATA 

# DNA-PK triggers histone ubiquitination and signaling in response to DNA double-strand breaks produced during the repair of transcription-blocking topoisomerase I lesions

## SUPPLEMENTARY DATA

- SUPPLEMENTARY DATA
