# Supplementary material for: DNA-PK triggers histone ubiquitination and signaling in response to DNA double-strand breaks produced during the repair of transcription-blocking topoisomerase I lesions
Source: Nucleic Acids Res. 2015 Nov 17;44(3):1161–78. doi: 10.1093/nar/gkv1196 (PMC4756817; doi:10.1093/nar/gkv1196)
Supplement: SUPPLEMENTARY DATA [file supp_gkv1196_nar-01852-d-2015-File010.pdf]

Supplementary Data

DNA-PK triggers histone ubiquitination and signaling in response to DNA double-strand breaks produced during the repair of transcription-blocking topoisomerase I lesions

Agnese Cristini, Joon-Hyung Park, Giovanni Capranico, Gaëlle Legube, Gilles Favre, Olivier Sordet

---

SUPPLEMENTARY FIGURE LEGENDS

Figure S1. Induction of γH2AX in quiescent WI38 hTERT cells in response to CPT. (A,B) Cells were cultured in 10% serum or in 0.2% serum for 3 days to induce quiescence. Cells were then exposed to 100 μM BrdU for 30 min before staining for BrdU (green). DNA was counterstained with DAPI (blue). **(A)** Representative images. Bars: 50 μm. **(B)** Percentages of BrdU-positive cells from one representative experiment (> 300 cells were analyzed for each treatment) out of two. **(C-E)** Serum-starved cells were treated with the indicated CPT concentrations for 1 h before staining for γH2AX (green). DNA was counterstained with DAPI (blue). **(C)** Representative images. Numbers are the percentage of nuclei with at least 2 γH2AX foci (bottom) and the average number of γH2AX foci per nucleus (top). Bars: 10 μm. **(D)** Number of γH2AX foci per nucleus. ****, P < 0.0001. **(E)** Quantification of γH2AX fluorescence intensity per nucleus. Quantifications in panels C to E are from two independent experiments (209-246 nuclei were analyzed for each treatment).

Figure S2. ATM- and DNA-PK-dependent induction of γH2AX in quiescent IMR90 and NHDF cells in response to CPT. Data in IMR90 cells are shown in panels A-D and data in NHFD cells are shown in panels E-H. **(A,E)** Cells cultured in 10% or 0.2% serum for 3 days were exposed to 100 μM BrdU for 30 min before staining for BrdU. Percentage of BrdU positive cells (> 300 cells were analyzed for each treatment). **(B-D, F-H)** Serum-starved cells were treated with DMSO or with ATMi (10 μM) or DNA-PKi (10 μM) for 1 h before the addition of DMSO (untreated) or CPT (25 μM) for 1 h. Cells were then stained for γH2AX (green) and DNA was counterstained with DAPI (blue). **(B,F)** Representative pictures. Numbers indicate the average number of γH2AX foci per nucleus. Bars: 10 μM. **(C,G)** Number of γH2AX foci per nucleus (122-167 nuclei were analyzed for each treatment in panel C and 111-169 in panel G); ****, P < 0.0001. **(D,H)** Quantification of γH2AX fluorescence intensity per nucleus (means ± SD, 182-209 cells were examined per condition in panel D and 208-267 in panel H).

Figure S3. Induction of transcription-dependent DSBs in quiescent WI38 hTERT cells in response to CPT. (A,B) Serum-starved cells were treated with DMSO or FLV (1 μM) for 1 h before the addition of DMSO (untreated) or CPT (25 μM) for 1 h and then co-stained for γH2AX (green) and 53BP1 (red). **(A)** Representative pictures. Images were merged to determine colocalization (yellow). Nuclear contours, identified by DAPI staining (not shown), are indicated by dashed lines. Bars: 10 μm. **(B)** Number of γH2AX foci per nucleus from one representative experiment (99-133 nuclei were analyzed for each treatment) out of four. ****, P < 0.0001. **(C,D)** Detection of DSBs by neutral Comet assays in serum-starved cells treated with DMSO or FLV (1 μM) for 1 h before the addition of DMSO (untreated) or CPT (7.5 μM) for 1 h. **(C)** Representative pictures of nuclei. **(D)** Quantification of neutral Comet tail moments from one representative experiment (101-106 nuclei were analyzed for each treatment) out of three. ****, P < 0.0001.

Figure S4. Top1 is degraded in response to CPT but not IR or *Asi*SI, and PARP inhibition increases γH2AX/53BP1 foci in CPT-treated quiescent cells. (A-C) Western blot of Top1 in serum-starved WI38 hTERT cells treated with DMSO or with FLV (1 μM, 1 h), G5 (1.5 μM, 0.5 h) or MG132 (10 μM, 1 h) before the addition of DMSO ("-" in panel A (4 h) and C (6 h); "untreated" in panel B) or CPT (25 μM) for the indicated times in panels A and C or for 4 h in panel B. **(D)** Western blot of Top1 and γH2AX in serum-starved WI38 hTERT cells 4 and 6 h post-irradiation with 0.8 Gy IR. **(E)** Western blot of Top1 and γH2AX in U2OS EV28 cells treated with ethanol ("-", 6 h) or 300 nM 4OHT for the indicated times. In panels A-E, Western blot have been performed in whole cell extracts allowing the detection of the whole cellular Top1 (Top1 and Top1cc) as previously reported (Ref. 13). αTubulin: loading control. **(F)** Schematic representation of the repair of a Top1cc. Top1 is partially proteolyzed

by the ubiquitin/proteasome system to expose the covalent bond between the Top1 catalytic tyrosine and the 3'-end of the DNA to be attacked by TDP1. Top1cc excision by TDP1 requires PARP1. TDP1 generates a 3'-phosphate, which is hydrolyzed by PNKP before religation by ligase III. **(G)** Western blot of TDP1 in serum-starved WI38 hTERT cells transfected with TDP1-targeting or nontargeting (Control) siRNAs. αTubulin: loading control. (**H,I**) Serum-starved WI38 hTERT cells were treated for 1 h with DMSO or olaparib (10 µM) before the addition of DMSO (untreated) or CPT (25 µM) for 1 h. Cells were then co-stained for γH2AX (green) and 53BP1 (red). **(H)** Representative pictures. Nuclear contours, identified by DAPI staining (not shown), are indicated by dashed lines. Bars: 10 µm. **(I)** Number of γH2AX foci per nucleus from one representative experiment (131-151 nuclei were analyzed for each treatment) out of two; ****, $P < 0.0001$.

Figure S5. Activation of ATM signaling and its prevention following DNA-PK inhibition in CPT-treated quiescent WI38 hTERT cells. (A,B) Serum-starved WI38 hTERT cells were treated with DMSO or with ATMi (10 µM) or DNA-PKi (10 µM) for 1 h before the addition of DMSO (untreated) or CPT (25 µM) for 1 h and stained for γH2AX (green). Representative pictures are shown in Figure 4A. **(A)** Quantification of γH2AX fluorescence intensity per nucleus (means ± SD, 172-307 nuclei were examined for each treatment). **(B)** Size of γH2AX foci (means ± SD, n = 3, > 320 foci from 50-70 cells were analyzed for each treatment in each experiment).**, $P < 0.01$; *t*-test. **(C)** Western blot of Chk1 phosphorylated on S345 (Chk1-pS345) and total Chk1 in replicating WI38 hTERT cells treated with DMSO or ATRi (10 µM) for 1 h before the addition of DMSO ("-", 4 h) or CPT (25 µM) for the indicated times. **(D)** Western blot of ATM, DNA-PK and ATR in WI38 hTERT cells cultured in 10% serum or in 0.2 % serum for 3 days. **(E)** Phosphorylation of ATM on S1981 (ATM-pS1981), KAP1 on S824 (KAP1-pS824), Chk2 on T68 (Chk2-pT68) and p53 on S15 (p53-pS15) was determined by Western blot in serum-starved WI38 hTERT cells treated with the indicated concentrations of CPT for 1 h. Total ATM, KAP1, Chk2 and p53 were examined in parallel. **(F)** Serum-starved WI38 hTERT cells were treated with DMSO or DNA-PKi (10 µM) for 1 h before the addition of DMSO (untreated) or CPT (25 µM) for 1 h and stained for 53BP1, 53BP1 phosphorylated on S1778 (p53BP1) or MDC1. Representative pictures from one experiment out of two. Nuclear contours, identified by DAPI staining (not shown), are indicated by dashed lines. Bars: 10 µm.

Figure S6. Inhibition of XLF or XRCC4 does not prevent the formation of ATM-pS1981 and γH2AX foci in CPT-treated quiescent WI38 hTERT cells. **(A,B)** Serum-starved WI38 hTERT cells were transfected with XLF-targeting or nontargeting (Control) siRNAs. **(A)** Western blot showing the efficiency of XLF silencing. Actin: loading control. **(B)** siRNA-transfected cells were treated with DMSO (untreated) or CPT (25 µM) for 1 h and pre-extracted with CSK buffer before co-staining for ATM-pS1981 (red) and γH2AX (green). Representative pictures. Nuclear contours, identified by DAPI staining (not shown), are indicated by dashed lines. Bars: 10 µm. **(C,D)** Similar experiments were performed following siRNA-mediated depletion of XRCC4.

Figure S7. Inhibition of DNA-PK or ATM but not XLF or XRCC4 prevents CPT-induced Top1 degradation in quiescent cells. (A) Western blot of H2AX in serum-starved WI38 hTERT cells treated with DMSO (untreated) or CPT (25 µM) for 1 h. +Ub1 indicates monoubiquitinated H2AX. **(B,C)** Serum-starved WI38 hTERT cells were transfected with DNA-PK-targeting or nontargeting (Control) siRNAs. **(B)** Western blot showing DNA-PK silencing. αTubulin: loading control. **(C)** siRNA-transfected cells were treated with CPT (25 µM) for 1 h before staining for ubiquitinated proteins (FK2). Number of FK2 foci per nucleus from two experiments (156-171 nuclei were analyzed for each treatment). ****, $P < 0.0001$, *t*-test. **(D)** Western blot of Top1 in serum-starved IMR90 cells (top) and serum-starved NHDF cells (bottom) treated with DMSO or DNA-PKi (10 µM) for 1 h before the addition of DMSO ("-", 6 h) or CPT (25 µM) for the indicated times. αTubulin: loading control. **(E)** Western blot of Top1, XLF and XRCC4 in serum-starved WI38 hTERT cells transfected with siRNAs against XLF, XRCC4 or a nontargeting sequence (Control) before treatment with DMSO (- CPT) or 25 µM CPT (+ CPT) for 6 h. Actin: loading control. **(F)** Western blot of Top1 in serum-starved WI38 hTERT cells treated with DMSO or ATMi (10 µM) for 1 h before the addition of DMSO ("-", 6 h) or CPT (25 µM) for the indicated times. αTubulin: loading control. In panels D-F, Western blot have been performed in whole cell extracts allowing the detection of the whole cellular Top1 (Top1 and Top1cc) as previously reported (Ref. 13). **(G)** Serum-starved WI38 hTERT cells were subjected to fractionation and proteins from whole cell extracts (WCE), 150 mM NaCl/0.1% Triton X-100 supernatant (S1), 150 mM NaCl/RNAse A supernatant (S2) and pellets (P2) were analyzed by Western blot. αTubulin and histone H3 control the S1 and P2 fractions, respectively. **(H)** DMSO (untreated), DNA-PKi (10 µM) or

MG132 (10 μM) were incubated with the P2 fraction of serum-starved WI38 hTERT cells and proteasome chymotrypsin-like activity was measured (means ± SEM, n = 3).

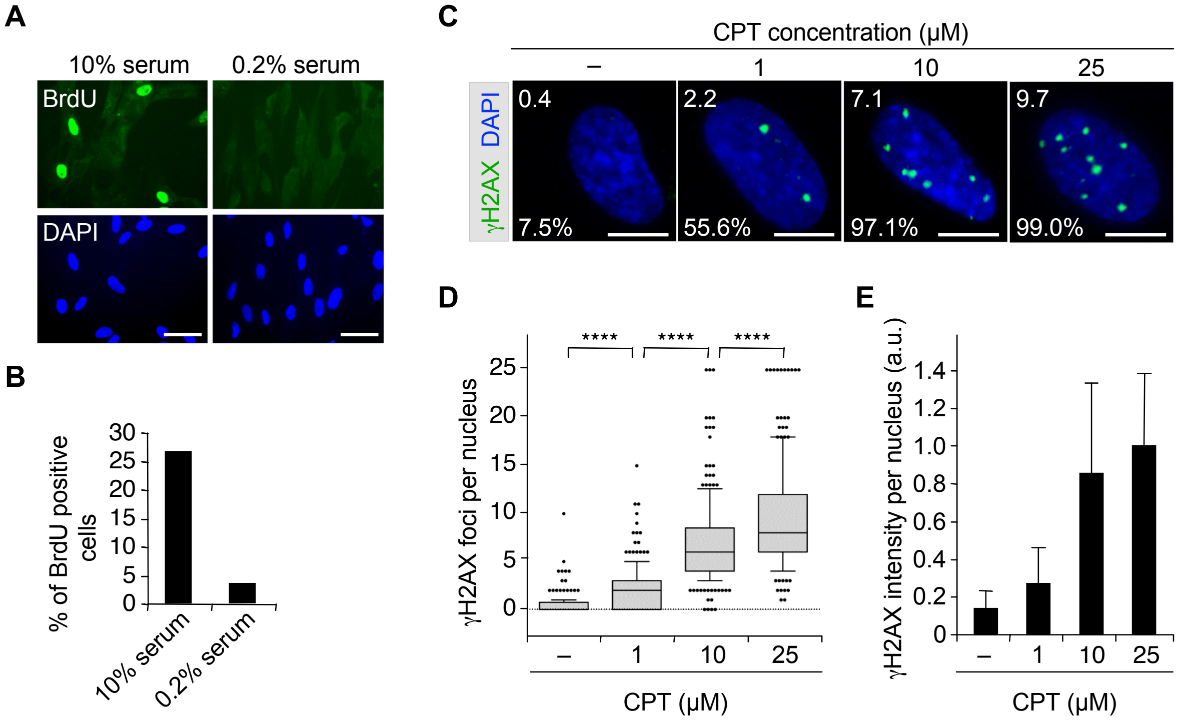
A
10% serum
0.2% serum
BrdU
DAPI
B
% of BrdU positive cells
30
25
20
15
10
5
0
10% serum
0.2% serum
C
CPT concentration (μM)
–
1
10
25
γH2AX DAPI
0.4
2.2
7.1
9.7
7.5%
55.6%
97.1%
99.0%
D
γH2AX foci per nucleus
****
****
****
25
20
15
10
5
0
–
1
10
25
CPT (μM)
E
γH2AX intensity per nucleus (a.u.)
1.4
1.2
1.0
0.8
0.6
0.4
0.2
0
–
1
10
25
CPT (μM)

Figure S1

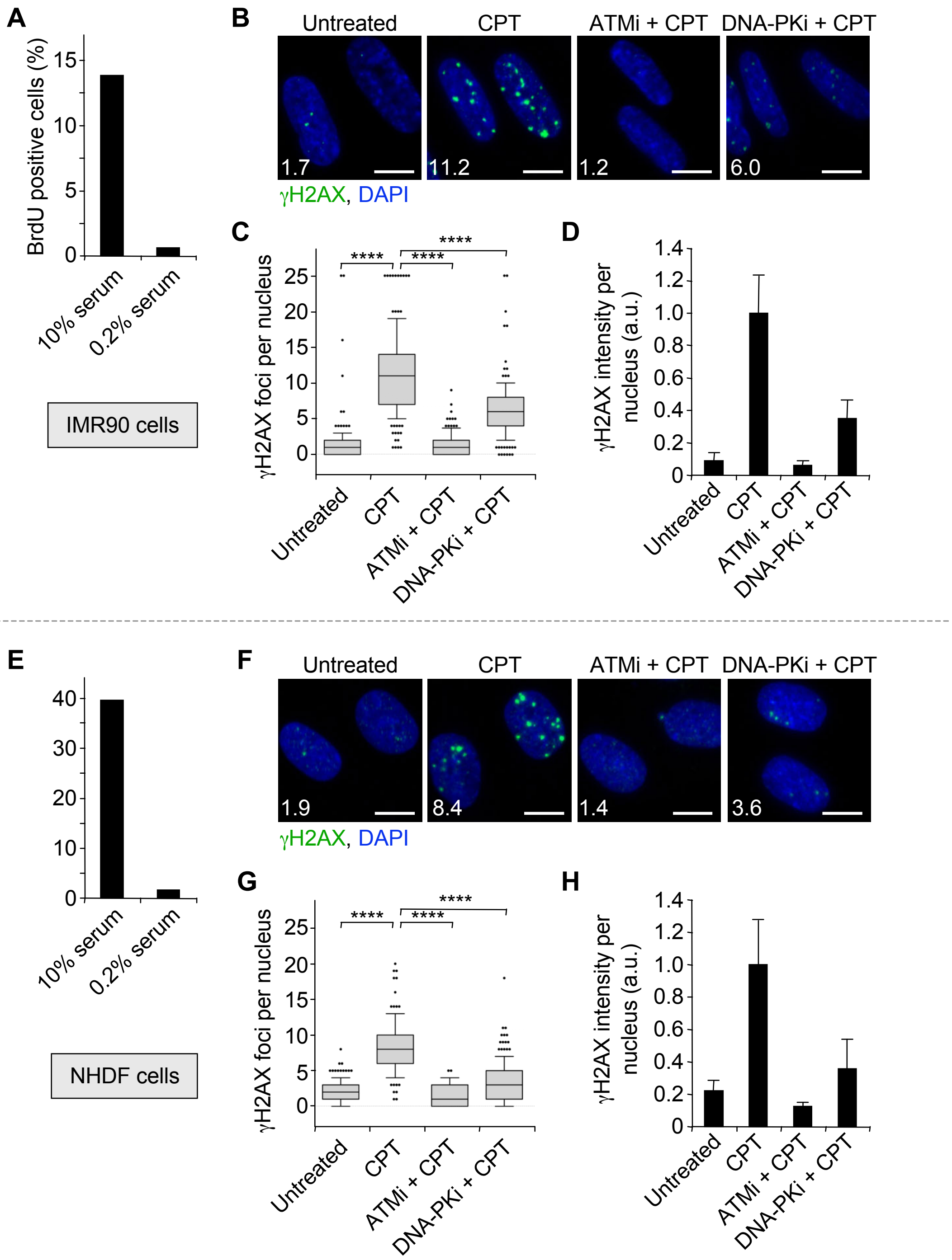
A
BrdU positive cells (%)
15
10
5
0
10% serum
0.2% serum
IMR90 cells
B
Untreated
CPT
ATMi + CPT
DNA-PKi + CPT
1.7
11.2
1.2
6.0
γH2AX, DAPI
C
γH2AX foci per nucleus
25
20
15
10
5
0
****
****
****
Untreated
CPT
ATMi + CPT
DNA-PKi + CPT
D
γH2AX intensity per nucleus (a.u.)
1.4
1.2
1.0
0.8
0.6
0.4
0.2
0
Untreated
CPT
ATMi + CPT
DNA-PKi + CPT
E
40
30
20
10
0
10% serum
0.2% serum
NHDF cells
F
Untreated
CPT
ATMi + CPT
DNA-PKi + CPT
1.9
8.4
1.4
3.6
γH2AX, DAPI
G
γH2AX foci per nucleus
25
20
15
10
5
0
****
****
****
Untreated
CPT
ATMi + CPT
DNA-PKi + CPT
H
γH2AX intensity per nucleus (a.u.)
1.4
1.2
1.0
0.8
0.6
0.4
0.2
0
Untreated
CPT
ATMi + CPT
DNA-PKi + CPT

Figure S2

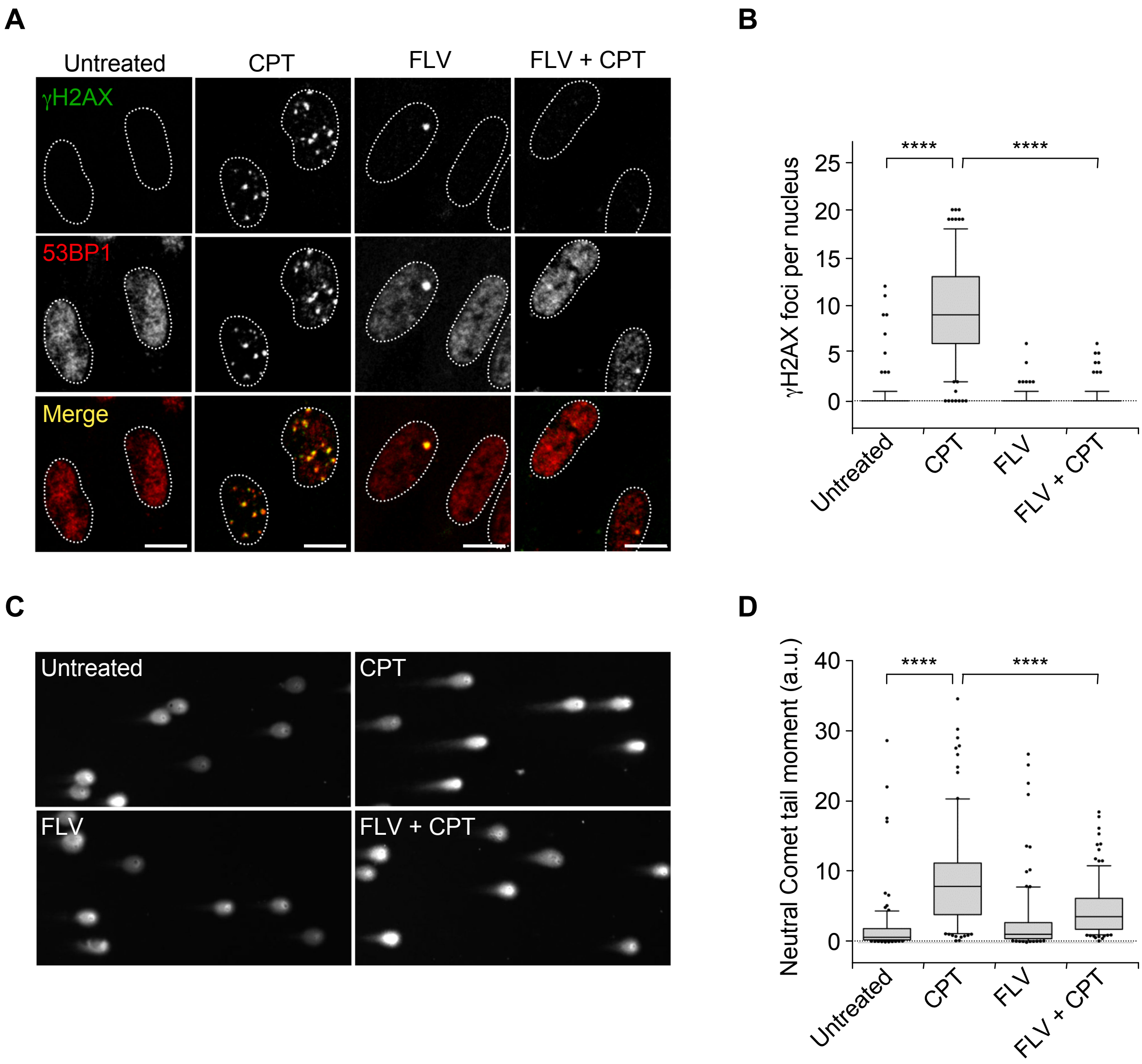
A
B
C
D
Untreated
CPT
FLV
FLV + CPT
γH2AX
53BP1
Merge
γH2AX foci per nucleus
25
20
15
10
5
0
****
****
Untreated
CPT
FLV
FLV + CPT
Neutral Comet tail moment (a.u.)
40
30
20
10
0

Figure S3

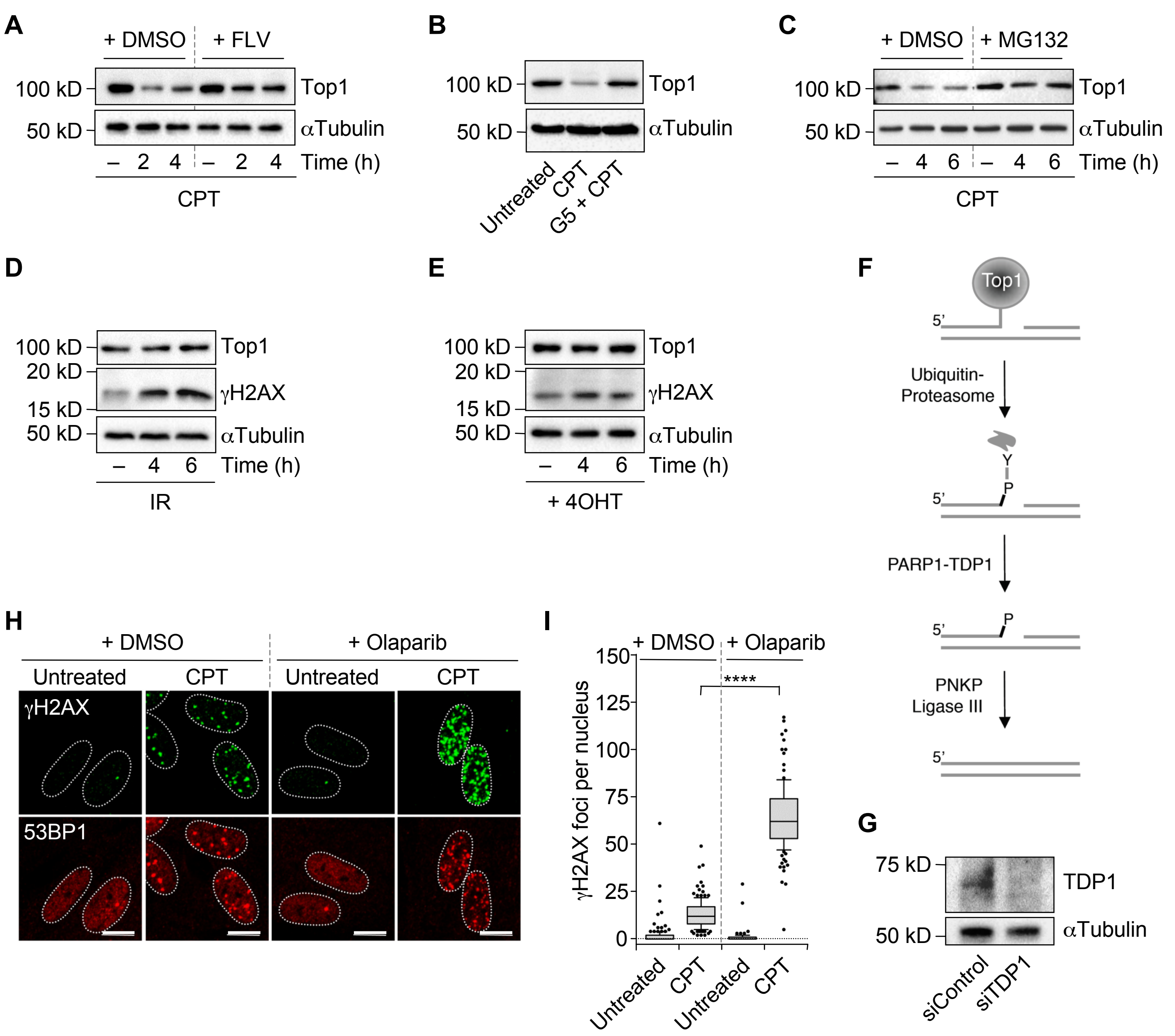

A
+ DMSO
+ FLV
100 kD
Top1
50 kD
αTubulin
– 2 4 – 2 4 Time (h)
CPT
B
100 kD
Top1
50 kD
αTubulin
Untreated
CPT
G5 + CPT
C
+ DMSO
+ MG132
100 kD
Top1
50 kD
αTubulin
– 4 6 – 4 6 Time (h)
CPT
D
100 kD
Top1
20 kD
γH2AX
15 kD
50 kD
αTubulin
– 4 6 Time (h)
IR
E
100 kD
Top1
20 kD
γH2AX
15 kD
50 kD
αTubulin
– 4 6 Time (h)
+ 4OHT
F
Top1
5'
Ubiquitin-
Proteasome
Y
P
PARP1-TDP1
PNKP
Ligase III
G
75 kD
TDP1
50 kD
αTubulin
siControl
siTDP1
H
+ DMSO
+ Olaparib
Untreated
CPT
Untreated
CPT
γH2AX
53BP1
I
+ DMSO
+ Olaparib
****
γH2AX foci per nucleus
150
125
100
75
50
25
0
Untreated
CPT
Untreated
CPT

Figure S4

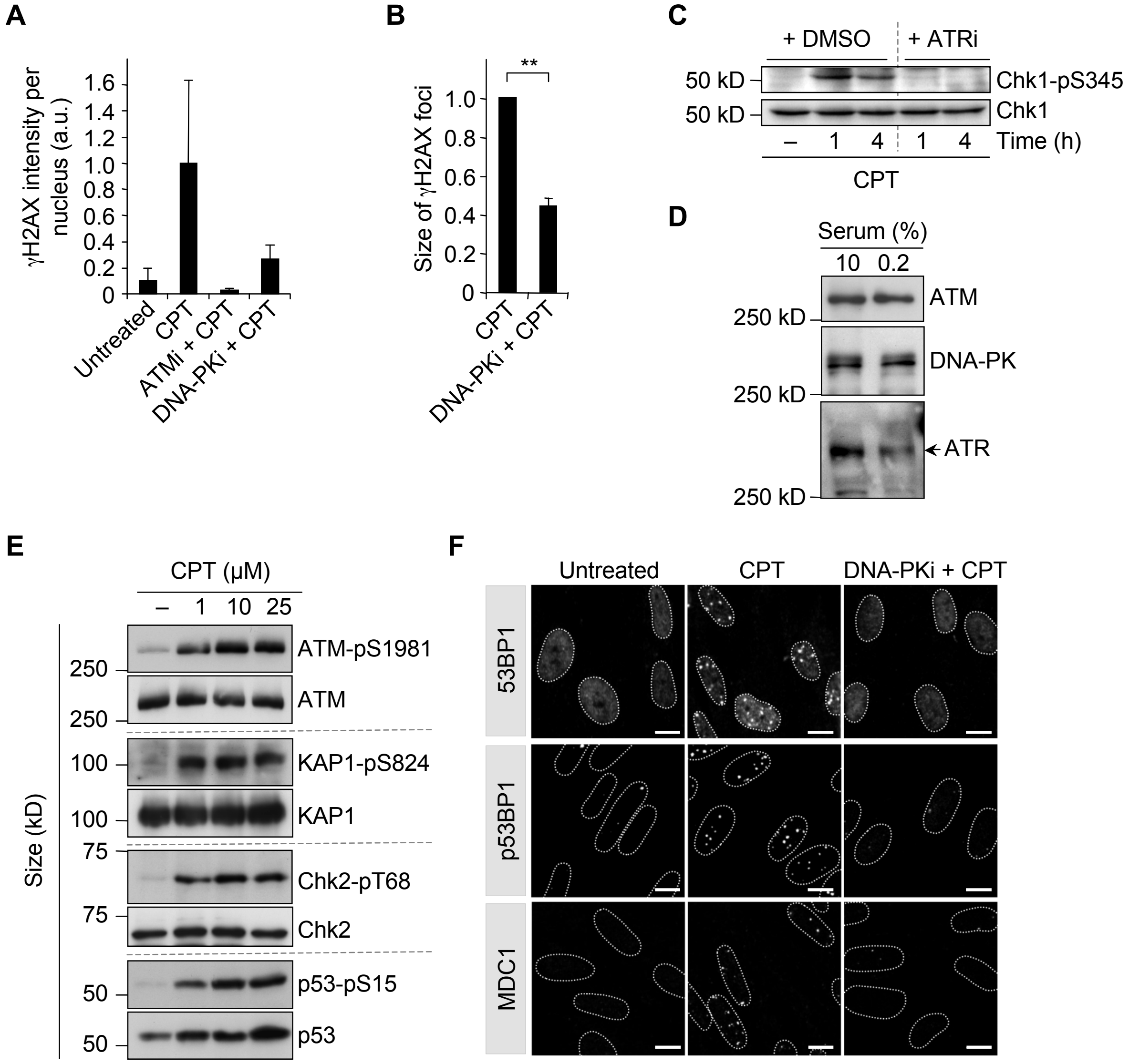
A
γH2AX intensity per nucleus (a.u.)
1.6
1.4
1.2
1.0
0.8
0.6
0.4
0.2
0
Untreated
CPT
ATMi + CPT
DNA-PKi + CPT
B
Size of γH2AX foci
**
1.0
0.8
0.6
0.4
0.2
0
CPT
DNA-PKi + CPT
C
+ DMSO
+ ATRi
50 kD
Chk1-pS345
50 kD
Chk1
–
1
4
1
4
Time (h)
CPT
D
Serum (%)
10
0.2
ATM
250 kD
DNA-PK
250 kD
ATR
250 kD
E
CPT (µM)
–
1
10
25
ATM-pS1981
250
ATM
250
KAP1-pS824
100
KAP1
100
75
Chk2-pT68
75
Chk2
p53-pS15
50
p53
50
Size (kD)
F
Untreated
CPT
DNA-PKi + CPT
53BP1
p53BP1
MDC1

Figure S5

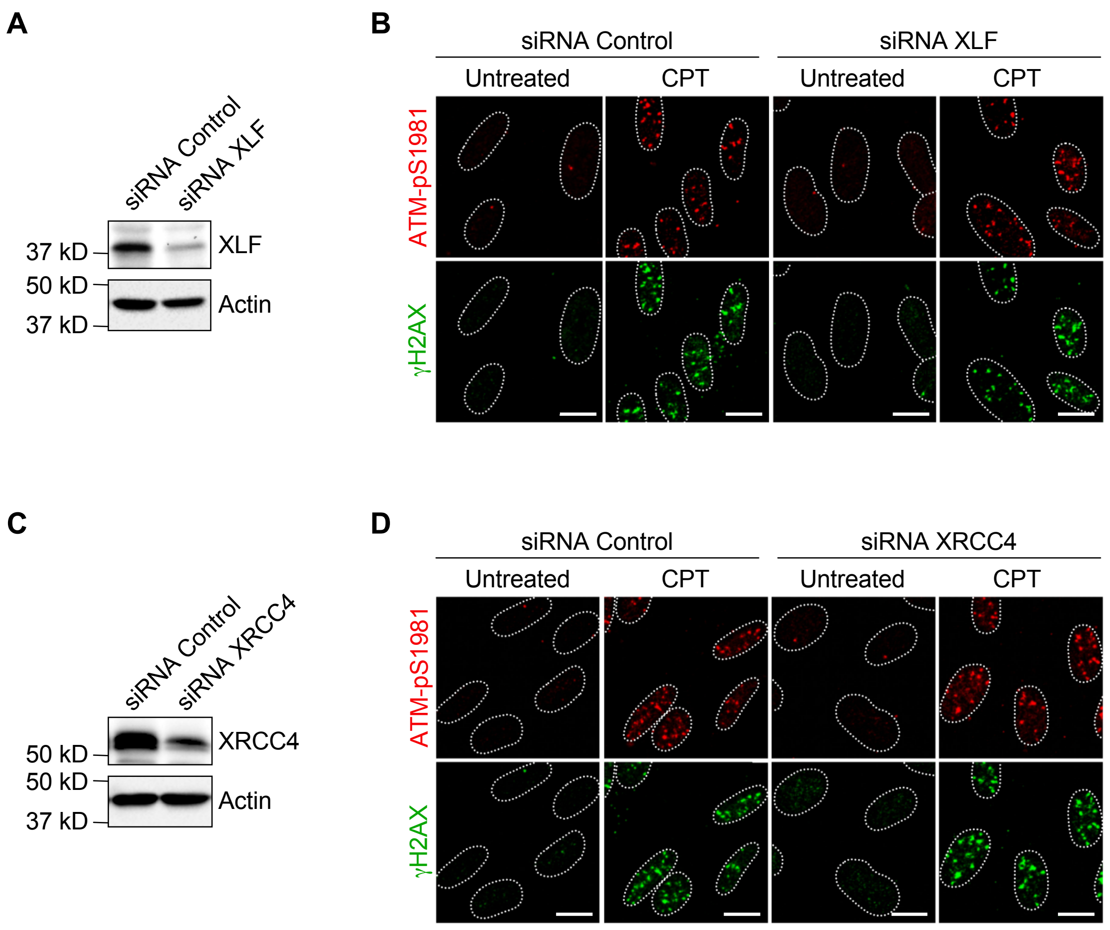
A
siRNA Control
siRNA XLF
37 kD
XLF
50 kD
Actin
37 kD
B
siRNA Control
siRNA XLF
Untreated
CPT
Untreated
CPT
ATM-pS1981
γH2AX
C
siRNA Control
siRNA XRCC4
50 kD
XRCC4
50 kD
Actin
37 kD
D
siRNA Control
siRNA XRCC4
Untreated
CPT
Untreated
CPT
ATM-pS1981
γH2AX

Figure S6

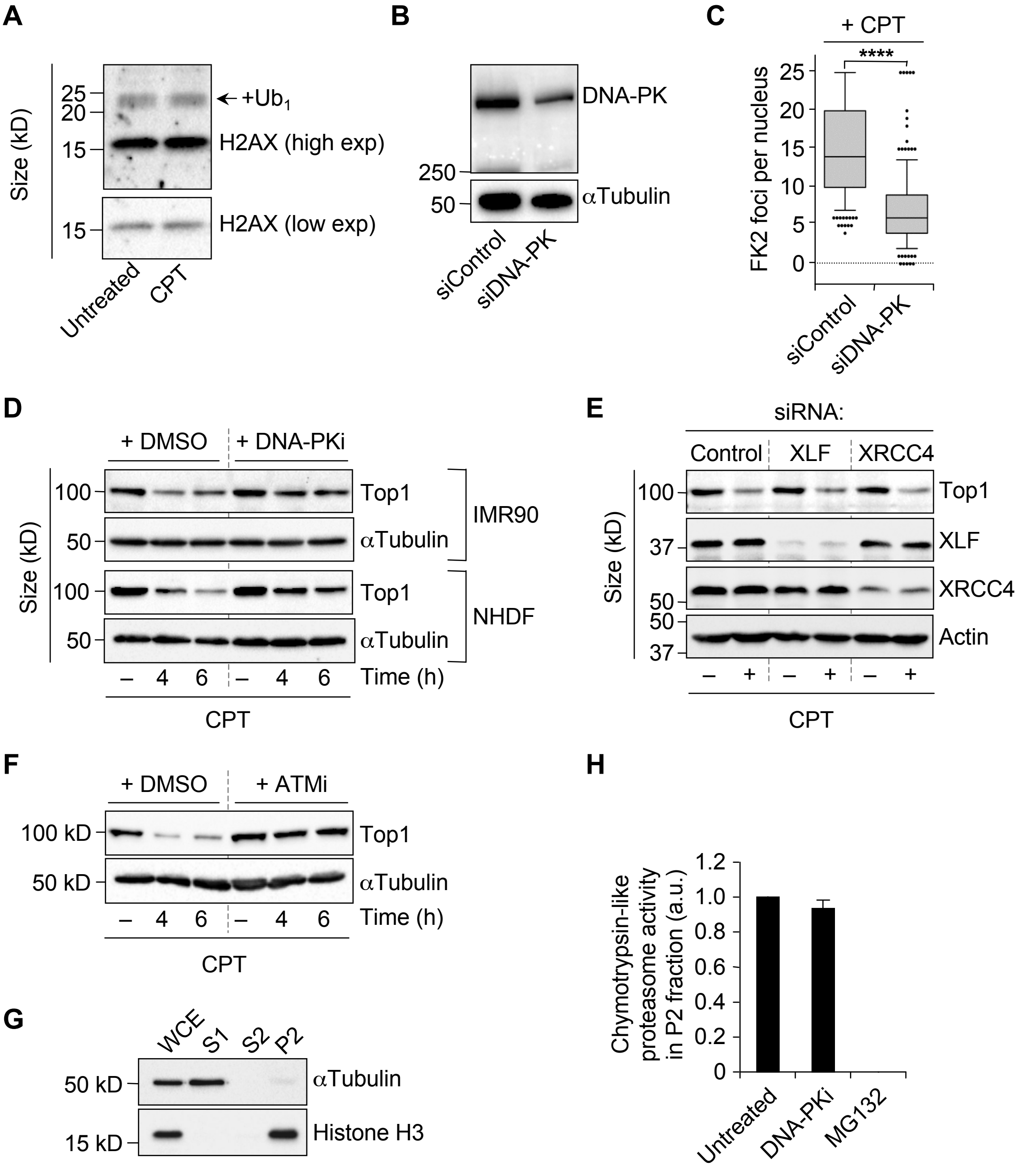

A
Size (kD)
25
20
15
←+Ub1
H2AX (high exp)
H2AX (low exp)
Untreated
CPT
B
DNA-PK
250
50
αTubulin
siControl
siDNA-PK
C
+ CPT
****
FK2 foci per nucleus
25
20
15
10
5
0
siControl
siDNA-PK
D
+ DMSO
+ DNA-PKi
100
50
Top1
αTubulin
IMR90
NHDF
– 4 6 – 4 6
Time (h)
CPT
E
siRNA:
Control
XLF
XRCC4
Top1
XLF
XRCC4
Actin
37
– + – + – +
CPT
F
+ DMSO
+ ATMi
100 kD
50 kD
Top1
αTubulin
Time (h)
CPT
G
WCE
S1
S2
P2
50 kD
15 kD
αTubulin
Histone H3
H
Chymotrypsin-like proteasome activity in P2 fraction (a.u.)
1.2
1.0
0.8
0.6
0.4
0.2
0
Untreated
DNA-PKi
MG132

Figure S7
